# Altered m6A RNA methylation contributes to hippocampal memory deficits in Huntington’s disease mice

**DOI:** 10.1007/s00018-022-04444-6

**Published:** 2022-07-11

**Authors:** Anika Pupak, Ankita Singh, Anna Sancho-Balsells, Rafael Alcalá-Vida, Marc Espina, Albert Giralt, Eulàlia Martí, Ulf Andersson Vang Ørom, Silvia Ginés, Verónica Brito

**Affiliations:** 1 Departament de Biomedicina, Facultat de Medicina, Institut de Neurosciències, Universitat de Barcelona, Casanova 143, 08036 Barcelona, Spain; 2 Institut d’Investigacions Biomèdiques August Pi i Sunyer (IDIBAPS), Barcelona, Spain; 3 Centro de Investigación Biomédica en Red Sobre Enfermedades Neurodegenerativas (CIBERNED), Madrid, Spain; 4 Department for Molecular Biology and Genetics, Aarhus University, Aarhus C, Denmark; 5 Laboratoire de Neurosciences Cognitives et Adaptatives (LNCA), University of Strasbourg, Strasbourg, France; 6 Centro de Investigación Biomédica en Red de Epidemiología y Salud Pública (CIBERESP), Madrid, Spain

**Keywords:** Huntington’s disease, m6A, Memory, Gene expression, Synaptic genes, Hippocampus, Post-transcriptional regulation, RNA chemical modifications

## Abstract

N6-methyladenosine (m6A) regulates many aspects of RNA metabolism and is involved in learning and memory processes. Yet, the impact of a dysregulation of post-transcriptional m6A editing on synaptic impairments in neurodegenerative disorders remains unknown. Here we investigated the m6A methylation pattern in the hippocampus of Huntington’s disease (HD) mice and the potential role of the m6A RNA modification in HD cognitive symptomatology. m6A modifications were evaluated in HD mice subjected to a hippocampal cognitive training task through m6A immunoprecipitation sequencing (MeRIP-seq) and the relative levels of m6A-modifying proteins (FTO and METTL14) by subcellular fractionation and Western blot analysis. Stereotaxic CA1 hippocampal delivery of AAV-shFTO was performed to investigate the effect of RNA m6A dysregulation in HD memory deficits. Our results reveal a m6A hypermethylation in relevant HD and synaptic related genes in the hippocampal transcriptome of *Hdh*^+*/Q111*^ mice. Conversely, m6A is aberrantly regulated in an experience-dependent manner in the HD hippocampus leading to demethylation of important components of synapse organization. Notably, the levels of RNA demethylase (FTO) and methyltransferase (METTL14) were modulated after training in the hippocampus of WT mice but not in *Hdh*^+*/Q111*^ mice. Finally, inhibition of FTO expression in the hippocampal CA1 region restored memory disturbances in symptomatic *Hdh*^+*/Q111*^ mice. Altogether, our results suggest that a differential RNA methylation landscape contributes to HD cognitive symptoms and uncover a role of m6A as a novel hallmark of HD.

**Supplementary Information:**

The online version contains supplementary material available at 10.1007/s00018-022-04444-6.

## Introduction

Huntington's disease (HD) is an autosomal dominant neurodegenerative disorder that typically develops in young-middle adulthood. This disease is characterized by choreiform movements that appear in the later, more advanced disease stages, and by cognitive deficits and psychiatric symptoms that often appear at early stages even before the onset of motor symptoms [1, 2]. Though these impairments are commonly attributed to corticostriatal dysfunction, accumulating evidence demonstrates that memory decline in HD is likely a reflection of a widespread brain circuitry defect that involves hippocampal dysfunction [3–5]. Accordingly, HD patients present alterations in associative learning, spatial short-term memory, spatial working memory and recognition memory, which all are known to involve the participation of the hippocampus and temporal lobe structure [3, 6, 7].

Studies in HD mouse models have revealed that these early cognitive deficits better associate with synaptic dysfunction rather than with neuronal cell loss [8–13]. To understand this neuronal dysfunction, these studies have focused on the control of gene expression at the transcriptional level by transcription factors or epigenetic modifications of DNA or histones [14–16]. However, post-transcriptional mechanisms such as the regulation of miRNA expression, mis-splicing and altered polyadenylation [17–21] have also been described to be critical in the regulation of gene expression in HD. Notably, all these processes can be regulated by RNA modifications, in particular N6-methyladenosine (m6A), the most abundant internal modification in mRNA that is especially enriched in the mammalian brain [22, 23].

The m6A mark can be dynamically incorporated in the RNA by a methyltransferase complex consisting of METTL3, METTL14 and WTAP [24, 25] and removed by the demethylases FTO and ALKBH5 [26–28]. Transcriptome-wide mapping of m6A revealed that this modification is mainly deposited at the DRACH (where D = A, G or U; H = A, C or U) consensus motif displaying a conserved pattern across mRNAs and lncRNAs [23, 29]. A range of m6A binding proteins have been reported to mediate multifaceted functions of mRNA metabolism including splicing, nuclear export, stability, translation, microRNA processing, and subcellular targeting of m6A-modified mRNAs [30–34]. In the mammalian brain, m6A is also important in the fine-tuning of the transcriptome during learning and memory consolidation, particularly in the hippocampus, striatum and prefrontal cortex [35–39]. Indeed, m6A has been found to be present in the synaptic transcriptome, selectively modifying a number of transcripts in all cellular domains of tripartite synapses [40]. The possibility that dysregulation of m6A modifications could contribute to the disruption of the mRNA metabolism in neuronal dysfunction has already been suggested; however, only few studies have addressed its importance in neurodegenerative disorders [41–46].

In the present study, we aimed to investigate the potential role of m6A RNA modifications in HD cognitive deficits. Our data indicate that m6A methylation changes occur in the RNA along HD progression and that dysregulated m6A RNA modifications are involved in HD cognitive disturbances. Taken together, our findings identify m6A perturbations as a novel mechanism involved in memory impairments in HD, proposing this RNA modification as a potential therapeutic target for HD treatment.

## Methods

### Animals

*Hdh*^*Q7/Q7*^* wild-type* mice and heterozygous *Hdh*^+*/Q111*^* knock-in* mice [47, 48] were bred and maintained on a C57BL/6 genetic background. *Hdh*^+*/Q111*^ mice present a targeted insertion of 109 CAG repeats in the murine *huntingtin* gene that extends the resulting polyglutamine segment to 111 residues. At 6 months of age, heterozygous mice present a HD-like phenotype with hippocampal long-term memory deficits, followed by motor coordination impairments at 8 months of age [13, 47]. To obtain age matched WT and *Hdh*^+*/Q111*^ littermates, male WT mice were crossed with female heterozygous *Hdh*^+*/Q111*^ mice. Only males from each genotype were used for the experimental procedures.

### Behavioral training

We trained 4–5- and 8-month-old WT and *Hdh*^+*/Q111*^ mice (pre-symptomatic and symptomatic disease stages, respectively) in the object location task (OLT) to induce hippocampal neuronal activity. Mice were first habituated to an open field in the absence of objects and spatial cues for two consecutive days (10 min/day). This open field consisted of a white open-top squared arena: 40 × 40 × 40 cm. The third day, mice were placed in the open field with two identical objects and four different black spatial cues on each wall. Mice were allowed to explore for 10 min and then returned to their home cage. 30 min after the training task, mice were sacrificed by cervical dislocation.

### Stereotaxic surgery and viral transduction in vivo

To knock down FTO expression, mice were stereotaxically injected with AVVs encoding eGFP scramble control shRNA or shFTO generated by Vector Biolabs. After anaesthesia with a mixture of oxygen and isoflurane, bilateral injection of AVVs (0.5 µl: 3.8 × 10^9^ GC/hemisphere) in the CA1 of the dorsal hippocampus was performed according to the following coordinates from the bregma (millimetres); antero-posterior − 2.0; Lateral ± 1.5, and dorsoventral: − 1.3 (CA1). Injection was carried out as previously described [13]. Animals were carefully monitored for 2 h and then returned to their home cage for 1 month. After this period, animals from the different experimental groups were subjected to behavioral assessment.

### Behavioral assessment

Animals were tracked and recorded with SMART Junior software (Panlab, Spain). Percentage of preference was calculated as (time exploring relocated object or new object)/(total time exploring both objects) × 100.

#### Object location task

OLT was performed as previously described [13]. During the acquisition phase on the third day, mice were presented with two identical objects (A and A’) placed in two adjacent corners of the experimental apparatus for 10 min, after which they were returned to their home cage. Twenty-four hours after the training phase, one of the objects was moved to the opposite corner, and mice were tested for 10 min.

#### Novel object recognition test

NORT was performed 3 days after the OLT as previously described [13]. Mice were presented with two identical objects on the third day (B and B´). After a delay of 24 h, animals were tested in the arena with a familiar object and a new object (B and C) placed in the same location, which they were allowed to explore for 10 min.

### RNA isolation

RNA from the hippocampus was extracted using the RNeasy® Lipid Tissue Mini Kit from Qiagen® (cat no. 74804), following the instructions of the manufacturer. Concentration of purified RNA eluted in nuclease-free H_2_O was measured using the Nanodrop 1000 spectrophotometer (Thermo Fisher Scientific®).

### Global m6A measurements

Total hippocampal m^6^A levels were assessed using the EpiQuik® m^6^A RNA Methylation Quantification Kit (Epigentek®, cat no. P-9005) and LC–MS/MS. EpiQuik was performed according to the manufacturer’s recommendations. RNA mass spectrometry was performed as previously described [49]. Mass spectrometric detection was performed using an Agilent 6495 Triple Quadrupole system.

### MeRIP-seq

m6A immunoprecipitation sequencing was performed following the refined protocol of Zeng et al*.* [50], with minor modifications. Briefly, 3 µg of total hippocampal RNA from 3 animals/condition were chemically fragmented to 150–200 nt. Ten percent of the fragmented RNA sample was set aside to serve as the input and the remainder was then used to proceed with the IP. For this purpose, magnetic Protein A Dynabeads® (Invitrogen® by Thermo Fisher Scientific®, cat no. 10002D) were tumbled for 6 h with 5 µg of rabbit polyclonal anti-m^6^A antibody (Millipore®, cat no. ABE572) in IP buffer at 4ºC. After incubation, the antibody–beads mixture was washed and incubated overnight with 500 µL of the IP reaction mixture containing 9 µL of the fragmented RNA, 100 µL of 5× IP buffer and 10µL of SUPERase-IN® RNase Inhibitor (Invitrogen® by Thermo Fisher Scientific, cat no. AM2696) with constant rotation at 4 ºC. The immunoprecipitated RNA was subjected to two rounds of competitive elution with an m6A containing buffer and the eluted RNA was then concentrated using the RNeasy® MinElute® Cleanup kit (Qiagen®, cat no. 74204). Library construction was performed following the instructions provided by the SMARTer® Stranded Total RNA-Seq kit v2- Pico Input Mammalian (Takara Bio®, cat no. 634412). Dual combinatorial indexes were assigned to each sample to allow for multiplexing. After normalization of the concentration, libraries were pooled volumetrically and sequenced on the NovaSeq platform [Illumina®, BGI (Hong Kong)] performing paired end sequencing (2 × 150 bp).

### MeRIP-seq data analysis

#### Quality control and adapter trimming

Coverage of the sequenced libraries was ~ 100 million reads (Q20% > 97 and Q30% > 93). Of those, the percentage of uniquely mapped reads was in the range of 65–85%, with 5% of multi-mapped reads. Adapter trimming was performed using Bbduk tool (BBtools—http://jgi.doe.gov/data-and-tools/bb-tools/), and the *adapter.fa* file obtained from BBTools (containing Illumina adapters). Additional trimming parameters were ktrim = r (indicating trimming from the right side), with Kmer size of 23, mink = 11 and hdist = 1. The command *tbo* was given to trim the adapter if it was based on pair overlap, and further *tpe* command allowed to trim both reads of the paired end sequencing to the same length.

#### Alignment of reads

Alignment of all the paired end reads with the mouse genome assembly mm9 was performed using STAR [51]. Samtools [52] view command was used to convert bam files to sam files. Further, to pick only uniquely mapped reads, Samtools view -q 255 filter was applied. Removal of repetitive regions was performed by filtering out the reads using bedtools intersect command [53].

#### Calculation of coverage

To count the reads mapping with all the overlapping features, quantification of the genes was performed using bedtools multicov. To obtain aligned reads, bam files belonging to all the replicates of an experimental condition were merged with the help of samtools merge command and reproducible regions were obtained from merged replicates.

#### Detection of m6A sites

Narrow peaks of each MeRIP/input sample pair were called using MACS2 [54] callpeak. We considered peaks that are present in at least two of the three replicates as reproducible, hence two out of three replicates were combined, using bedtools and irreproducible discovery rate (IDR). Further, IDR [55] was used to check the reproducibility between the replicates taking p-value as a ranking measure. Combined peaks were filtered to find significant peak calls using IDR score filter (< = 0.05). Coordinates of all of the reproducible peaks in combination of two were concatenated and later collapsed using mergeBed [53]. Annotation of the reproducible merged peaks with the *gencode.vM1.annotation.gtf* file was performed using intersectBed [53].

#### Differential peak analysis, annotation and enrichment analysis

Differential peak analysis was performed between conditions with the reproducible peaks, using a DiffBind tool (annotationdbi) [56]. Statistically differential peak-sets were obtained using DESeq2 and filtered using *p* value < 0.05. Adjusted p-value was obtained by Benjamin and Hochberg multiple testing adjustment. Further, annotation of the differential peaks to the corresponding genes was performed with bedtools intersect using *mm9.knownGene.bed*. Annotation of gene biotypes (3′/5′ UTR, CDS exons and introns) of differentially bound peaks was performed using ChIPseeker [57].

#### Gene ontology

For gene ontology (GO), significant differential peaks were analyzed with Ingenuity Pathway Analysis (IPA) software (QIAGEN—http://www.ingenuity.com) and Synaptic Gene Ontologies (SynGo) [58].

#### Motif identification

Motif analysis was run de novo using HOMER (version 4.11) [59], considering ± 100 nucleotide intervals around the peak summit of ~ 3500 best scoring reproducible peaks in the 3’UTR. Background sequences were generated from respective input sequences using the scrambleFasta.pl script. Next, de novo motif search was run using findMotifs.pl script with -rna -p 10 -len 6 as parameters.

#### Differential gene expression

For analysis of RNA expression, readcounts from input samples were used applying a CPM cutoff of 1 or above in all three biological replicates [60] to discriminate expressed genes for the entire dataset. Genes were normalized using TMM algorithm [60] and calcNormFactor of edgeR [61]. The relationship function voom [62] from the limma package was used to establish the mean variance relationship and generate weights for each observation. The lmFit function of limma was used to transform the RNA-Seq data before linear modeling and find differentially expressed (DE) genes.

### Subcellular fractionation and western blot analysis

Nuclear enrichment was performed as described previously [13]. Enrichment of the nuclear fraction was assessed by the presence of the specific nuclear marker Histone H3.

Synaptosomal fractionation was performed as described elsewhere in Bruyere et al. [63]. Enrichment of each fraction was assessed by the presence or absence of specific synaptosomal markers: synaptophysin (non-PSD fraction) and postsynaptic density protein 95 (PSD95) (PSD fraction).

A standard Western blot protocol was employed to quantify protein levels from the subcellular fractionations with the following antibodies: FTO (1:1000, NovusBio, cat nº NB110-60,935), METTL14 (1:2000, Sigma-Aldrich, cat nº HPA038002), PSD-95 (1:1000, Cell Signalling Technology, cat no. 3450), synaptophysin (1:1000, Synaptic Systems, cat no. 101011), Histone H3 (1:1000, Cell Signaling Technology). Immunoreactive bands were developed by the enhanced chemiluminiscence method, detected using ChemiDoc imaging system (Bio-Rad®) or films. ImageLab® Software Version 6.0 (2017) or Image J were used for quantification.

### Tissue fixation, immunofluorescence and confocal imaging

Animals were deeply anaesthetized and immediately perfused intracardially with 4% (weight/vol) paraformaldehyde in 0.1 M phosphate buffer. Immunofluorescence was performed as previously described [13]. Following permeabilization and blocking, free floating brain sections were incubated overnight at 4 ºC with the primary antibodies against FTO (1:200, NovusBio, cat no. NB110-60935) and m6A (1:200, Synaptic Systems, cat no. 202 111). Samples were incubated with appropriate secondary antibodies (1:200, Cy3-coupled fluorescent secondary antibody, Jackson ImmunoResearch Laboratories, cat no. 715-165-150; 1:200, Alexa Fluor 647 goat anti-mouse, Invitrogen, cat no. A21236) for 2 h at room temperature. Nuclei were stained for 10 min with 4′,6-diamidino-2-phenylindole (DAPI) (1:5000, Sigma Aldrich, cat nº D9542). Representative images from the CA1 nuclear layer were obtained with a Leica SP5 laser scanning confocal microscope (Leica). Integrated optical density of nuclear FTO was analyzed with Image J.

### Statistical analysis

Raw data were processed using Excel® Microsoft Office and for further analysis transferred to Graphpad Prism® version 8.0.2 for Windows. Results are expressed as mean ± SEM. Normal distribution was assessed with the Shapiro–Wilk test. For statistical analysis, unpaired Student’s t-test (two-tailed) or two-way ANOVA was performed, and the appropriate post-hoc tests were applied as indicated in the figure legends. A 95% confidence interval was used, considering differences statistically significant when *p* < 0.05.

## Results

### Landscape of m6A in the ***Hdh***^+***/Q111***^ mouse hippocampus under naive and cognitive-trained conditions

**Fig. 1:**
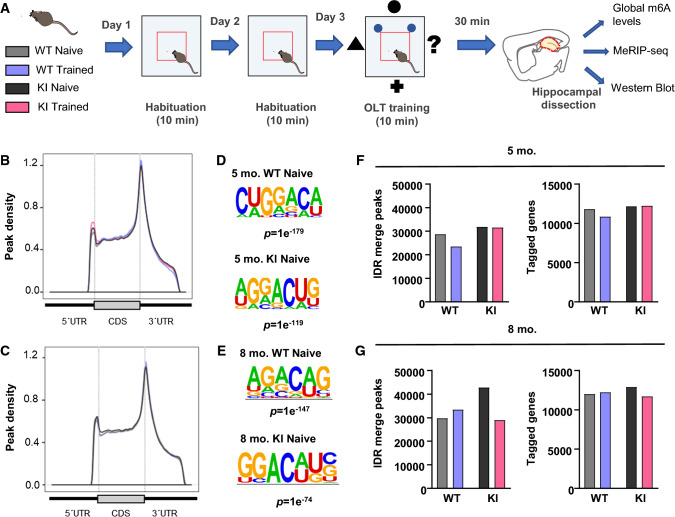
Mapping of the transcriptome-wide m6A landscape in the hippocampus of WT and Hdh^+/Q111^ (KI) mice. **A** Schematic illustration of the experimental design used to induce hippocampal activity. Mice were habituated to the open-field arena for two consecutive days. At the third day mice were trained on the OLT for 10 min and sacrificed after 30 min for hippocampal dissection to perform global m6A measurement, MeRIP-seq and Western Blot analysis. Metagene plot showing the m6A peak distribution along the mRNA in 5- (**B**) and 8- (**C**) month-old mice. Peaks are enriched at the CDS and 3′UTR in all conditions. Sequence logo representing the deduced consensus motif associated with m6A peaks identified by HOMER in 5- (**D**) and 8- (**E**) month-old mice. Histograms depict the number of reproducible m6A peaks in each condition (left) and the number of m6A modified RNAs (right) in 5- (**F**) and 8- (**G**) month-old mice. *IDR* irreproducible discovery rate, *KI* knock-in

Previous studies have shown that dynamic regulation of m6A on mRNA confers transcriptome plasticity while altered m6A methylation severely impacts gene expression and impairs learning and memory [35–40]. Therefore, we sought to evaluate whether HD mutation alters the m6A profile on mRNA under naive and cognitive training conditions in *Hdh*^+*/Q111*^ heterozygous mice. Behavioral analysis of mice subjected to the training session in the spatial task (Fig. 1a) showed no significant differences between genotypes at 5 months of age while at 8 months of age *Hdh*^+*/Q111*^ mice reproduced the well-described defects in cognition and motor coordination (Supplementary Fig. 1a–f) [13, 47]. Next, global m6A levels were assessed in total hippocampal RNA from naive mice by liquid chromatography-tandem mass spectrometry (LC–MS/MS). We found increasing m6A levels in hippocampal RNA from *Hdh*^+*/Q111*^ mice when compared to WT mice (Supplementary Fig. 2a). Similar upregulation under naive conditions was observed when m6A levels were further measured using the EpiQuik m6A RNA Methylation Quantification Kit. Interestingly, we observed that following training, similar m6A levels were observed in WT mice compared to naive conditions while a significant decrease was found in *Hdh*^+*/Q111*^ mice (Supplementary Fig. 2b).

To further identify and characterize the m6A RNA modification pattern, m6A RNA immunoprecipitation and sequencing (MeRIP-seq) was performed. The distribution of the peaks is represented as a metagene plot in Fig. 1b and c, showing a similar enrichment of m6A density at the coding sequence (CDS) and 3′-untranslated regions (UTR) in all conditions. Analysis of the sequence context around called peaks showed enrichment of the m6A consensus site DRACH in the different conditions analyzed (Fig. 1d and e). These data indicate that our m6A profiling results are in accordance with the published m6A features [23, 29]. MeRIP-seq analysis identified around 30,000 m6A peaks for each of the conditions corresponding to around 11,000 m6A modified mRNAs (Fig. 1f and g; Supplementary table 1). Consistent with our findings showing increased m6A levels in RNA from naive symptomatic *Hdh*^+*/Q111*^ mice (Supplementary Fig. 2), the number of m6A peaks in these mice was 1.4-fold higher compared to WT mice (Fig. 1f and g).

### Altered transcriptome methylation in ***Hdh***^+***/Q111***^ mouse hippocampus affects synaptic and HD-related genes at pre- and symptomatic disease stages

**Fig. 2:**
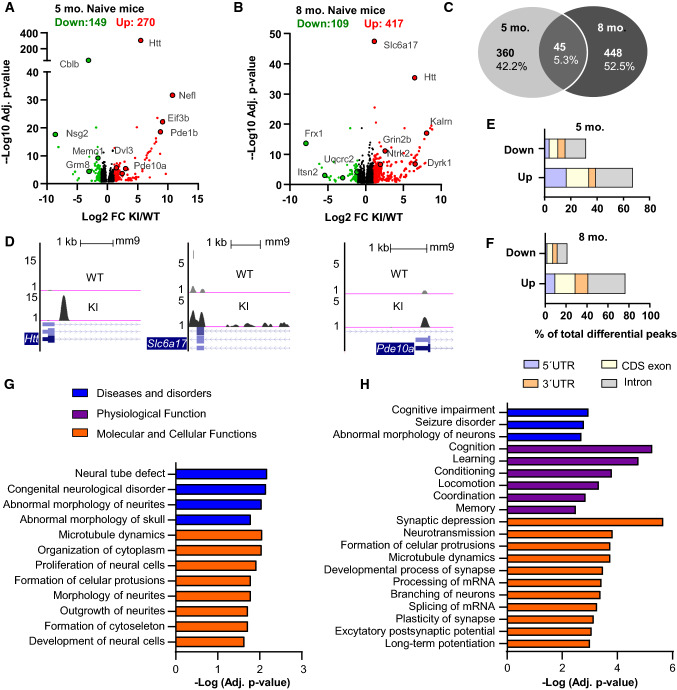
Mutant huntingtin induces alterations in hippocampal m6A RNA modifications affecting synaptic and HD-related genes. Volcano plots of the differentially methylated peaks between naive KI and WT animals at 5- (**A**) and 8- (**B**) months of age. Names of representative genes are indicated. **C** Venn diagram of the overlap of differential m6A modified transcripts between naive KI and WT mice at 5- and 8- months of age. Only 5% of differential m6A marked transcripts overlap between pre- and symptomatic disease stages. **D** UCSC Genome browser tracks of representative differential m6A peaks along indicated mRNAs. The y-axis represents normalized number of reads. Blue thick boxes represent the open reading frame while the blue line represents the untranslated regions (arrows point to the direction of transcription). Grey reads are from WT animals, black reads are from KI animals. Scale bar 1 kb. Distribution analysis of the down- and up-regulated differential m6A peaks between naive KI and WT mice at 5- (**E**) and 8- (**F**) months of age along the four transcript segments: 5′UTR, CDS exon, 3’UTR, intron. Data are shown as percentage of total differential peaks. IPA of differentially hypermethylated transcripts between naive KI and WT mice at 5- (**G**) and 8- (**H**) months of age. Bar plots showing the -Log (Adj. *p* value) of enriched terms belonging to the different categories. Screening threshold: Adjusted *p* value < 0.05 and log2 fold change > 1 or < − 1. *FC* fold change

In order to characterize basal m6A methylation signatures along the disease progression, we analyzed the differential m6A methylation peaks between naive WT and *Hdh*^+*/Q111*^ mice by a differential enrichment analysis at 5 and 8 months of age (Supplementary Tables 2 and 3). Adjusted *p* value < 0.05 and log2fold change > 1 or < − 1 were used as screening threshold of differential peaks. At 5 months of age, 270 peaks were hypermethylated and 149 peaks were hypomethylated in *Hdh*^+*/Q111*^ mice when compared to WT mice, while at 8 months of age 417 peaks were hypermethylated and 109 hypomethylated (Fig. 2a and b). When comparing all the differential peaks observed at pre-symptomatic and symptomatic stages, we identified only 5% of overlapping m6A tagged genes, while 42% and 53% of the differential methylated genes were unique for each stage (Fig. 2c), indicating a unique pattern of pathological methylation at each disease stage. To investigate the association between m6A methylation and mRNA expression, we intersected differential mRNA expression with differential m6A peaks. At 5 months of age, we found a greater number of m6A-containing mRNAs with significant differences in mRNA expression (47%) than at 8 months of age (23%) (Supplementary Fig. 3a), although no significant correlation between down and upregulation of mRNA species and m6A enrichment was found (Supplementary Fig. 3b and c). At both stages, we identified changes in the levels of m6A in different genes linked to HD, such as *Pde10a* and *Eif3b* (at 5 months), *Kalrn*, *Ntrk2*, *Gnaq*, *Grin2b*, *Dyrk1a* (at 8 months) and *Htt* itself (at 5 and 8 months), thus strengthening our hypothesis that altered m6A methylation plays a role in HD pathology (Tables 1 and 2). We also detected significant changes in m6A methylation in synaptic genes such as *Nefl* (at 5 months) and *Slc6a17*, *Frx1* (at 8 months) (some representative genes are shown in Fig. 2d).

**Table 1:** Differential m6A peaks in genes linked to HD in 5-month-old naive mice

Gene name	Gene symbol	Log2FC	Adjusted *p* value	Peak width	PMID
Eukaryotic translation initiation factor 3 subunit b	*Eif3b*	9.12	7.18E−23	424	25,959,826
Phosphodiesterase 1b	*Pde1b*	8.76	2.65E−19	585	14,751,289
Huntingtin	*Htt*	5.52	1.28E−303	502	6,316,146
Limbic system associated membrane protein	*Lsamp*	4.75	0.00623	537	17,500,595
Glutamate metabotropic receptor 4	*Grm4*	3.03	4.06E−06	351	29,643,462, 21,177,255
Caspase 9	*Casp9*	1.99	1.73E−05	368	12,095,160
NALCN channel auxiliary factor 1	*Fam155a*	1.68	0.00165	895	34,721,539
Calcium voltage-gated channel auxiliary subunit beta 4	*Cacnb4*	1.58	0.00058	566	29,936,182
Protein tyrosine phosphatase receptor type f	*Ptprf*	1.42	0.0435	240	27,378,699, 22,748,968
Integrin subunit alpha 7	*Itga7*	1.39	0.0417	326	29,328,442
Twist family BHLH transcription factor 1	*Twist1*	1.36	0.00422	612	29,891,550, 31,813,126
Potassium voltage-gated channel interacting protein 4	*Kcnip4*	1.33	0.00275	228	34,721,539, 22,965,876
Phosphodiesterase 10a	*Pde10a*	1.32	0.0499	694	26,198,591, 15,610,167
CUGBP elav-like family member 2	*Celf2*	1.26	0.00369	402	22,848,491
Sortilin related VPS10 domain containing receptor 2	*Sorcs2*	1.19	0.0119	426	28,469,074
F-box protein 41	*Fbxo41*	1.14	0.048	288	34,151,850
Inhibitor of nuclear factor kappa b kinase regulatory subunit gamma	*Ikbkg*	− 1.05	0.0487	318	26,949,515
Fas cell surface death receptor	*Fas*	− 1.07	0.000394	586	11,054,182, 25,800,750
Dihydropyrimidine dehydrogenase	*Dpyd*	− 1.13	0.00313	395	34,233,199
Contactin 1	*Cntn1*	− 1.27	0.000166	277	33,305,259, 30,554,964
Glutamate ionotropic receptor AMPA type subunit 3	*Gria3*	− 1.27	0.00785	262	24,211,138
Potassium voltage-gated channel interacting protein 4	*Kcnip4*	− 1.38	0.00251	229	34,721,539, 22,965,876
Solute carrier family 1 member 3	*Slc1a3*	− 1.38	0.0208	298	32,070,434
Caveolin 1	*Cav1*	− 1.61	7.52E−14	849	24,021,477

Table showing the differential m6A peaks in 5-month-old naive mice (KI vs WT) in genes that have been linked to Huntington’s disease. PubMed identifiers (PMID) for references supporting the link to HD are indicated. *FC* fold change

**Table 2:** Differential m6A peaks in genes linked to HD in 8-month-old naive mice

Gene name	Gene symbol	Log2FC	Adjusted *p* value	Peak width	PMID
Kalirin rhoGEF kinase	*Kalrn*	8.07	8.56E−18	239	26,464,483
G protein subunit alpha q	*Gnaq*	7.05	2.31E−06	705	27,924,190
Glutamate ionotropic receptor NMDA type subunit 2b	*Grin2b*	6.83	5.30E−06	213	21,989,477, 17,569,088
Dual specificity tyrosine phosphorylation regulated kinase 1a	*Dyrk1a*	6.57	1.47E−07	318	15,906,374
Huntingtin	*Htt*	6.49	3.91E−36	491	6,316,146
Calpain 5	*Capn5*	5.08	0.00263	205	14,981,075
Integrin subunit alpha 7	*Itga7*	3.85	0.00139	317	29,328,442
Proteasome 26S subunit, non-ATPase 13	*Psmd13*	3.09	2.02E−08	484	25,959,826
Phospholipase c beta 1	*Plcb1*	2.59	0.0286	228	17,519,223
Jumonji domain containing 6, arginine demethylase and lysine hydroxylase	*Jmjd6*	2.19	0.0378	311	25,927,346
Hexose-6-phosphate dehydrogenase	*H6pd*	1.99	1.73E−05	691	25,761,110
Neurotrophic receptor tyrosine kinase 2	*Ntrk2*	1.96	1.93E−07	193	16,487,146
Proteasome 26S subunit, non-ATPase 3	*Psmd3*	1.9	0.0093	569	25,910,212
LDL receptor related protein 1b	*Lrp1b*	1.45	0.000134	752	34,233,199
Protein kinase c epsilon	*Prkce*	1.45	0.00331	184	15,815,621
Histone deacetylase 8	*Hdac8*	1.36	0.00474	233	25,535,382
Caveolin 1	*Cav1*	1.32	1.27E-05	357	24,021,477
Growth factor receptor bound protein 2	*Grb2*	1.29	3.95E-09	364	24,116,161
Ryanodine receptor 2	*Ryr2*	1.22	0.0306	299	32,897,880
Calcium/calomodulin dependent protein kinase 2 beta	*Camk2b*	1.21	0.0232	196	30,451,379
Glutamate ionotropic receptor delta type subunit 2	*Grid2*	1.06	0.0275	287	29,480,208
Limbic system associated membrane protein	*Lsamp*	− 1.16	7.02E−05	431	17,500,595
Limbic system associated membrane protein	*Lsamp*	− 1.18	0.00056	912	17,500,595
Kalirin rhoGEF kinase	*Kalrn*	− 1.39	0.0123	387	26,464,483
Ubiquinol-cytochrome c reductase core protein 2	*Uqcrc2*	− 3.04	0.00553	411	17,500,595

Table showing the differential m6A peaks in 8-month-old naive mice (KI vs WT) in genes that have been linked to Huntington’s disease. PubMed Identifiers (PMID) for references supporting the link to HD are indicated. *FC* fold change

Since the unique distribution of m6A along the transcripts provides hints as to its functions [23, 29], we determined the location of the up and downregulated m6A peaks in *Hdh*^+*/Q111*^ mice at 5 and 8 months of age. We divided the transcript into four areas: 5'UTR, 3′UTR, CDS exon, and intron (Fig. 2e and f). All the differential m6A peaks showed similar distribution at 5 and 8 months, with a slight but not significant increase of upregulated peaks in the 3′ UTR and a decrease of upregulated peaks in the 5´UTR at 8 months compared to 5-month-old mice (Supplementary Fig. 3d). Moreover, we found around 40% of the m6A peaks located in introns. Since m6A changes may also influence RNA splicing and polyadenylation [64–67], we next explored whether the observed alterations in methylation in *Hdh*^+*/Q111*^ mice could be related with known genes affected by mis-splicing or aberrant polyadenylation in HD [18, 21]. We integrated our dataset with published datasets of alternative splicing and polyadenylation analysis from the R6/1 HD mouse model at symptomatic disease stages (3,5 and 7–8 months, respectively) [18, 21]. Integration analysis showed a significant over-enrichment of genes with changes in m6A levels and genes described to be mis-spliced, both at 5 and 8 months of age (Supplementary Fig. 4a, b and Supplementary Tables 4 and 5). Notably, the majority of the m6A peaks present in overlapping methylated and miss-spliced genes were found in introns and exon (Data not shown). At 8 months, we also detected a significant overlap between genes with m6A changes and genes described to have an altered poly(A) tail length in 7–8 month-old R6/1 mice (Supplementary Fig. 4c and d and Supplementary Tables 6 and 7). These results suggest that alteration of m6A methylation in hippocampal genes could be associated to aberrant alternative splicing and/ or polyadenylation in HD.

To analyze whether these changes in the m6A landscape along disease progression are associated to distinct functional signatures, we performed Ingenuity Pathway Analysis (IPA) with the lists of all transcripts differentially hypo- or hypermethylated between *Hdh*^+*/Q111*^ and WT mice at 5 and 8 months of age. Top diseases and bio functions obtained with IPA are given in Supplementary Fig. 5a–d. IPA for diseases and disorders revealed an enrichment in neurological diseases specifically in the hypermethylated transcripts, both at 5 and 8 months of age. Likewise, among molecular and cellular functions, terms related to cells morphology including “microtubule dynamics” and “formation of cellular protrusions,” were particularly enriched in hypermethylated transcripts at 5 months of age, while “synaptic depression,” “neurotransmission,” “formation of cellular protrusions” as well as “processing of mRNA,” among others, were enriched in hypermethylated transcripts at 8 months (Fig. 2g, h and Supplementary Tables 8 and 9 for complete lists of all biological functions and their associated genes). The top physiological system development and functions at 8 months of age were “cognition,” “learning” and “locomotion.” These processes have been described to be influenced by mutant huntingtin toxicity [2, 68–72] and our findings suggest that this is in part mediated by altered regulation of m6A post-transcriptional mRNA modifications.

**Fig. 3:**
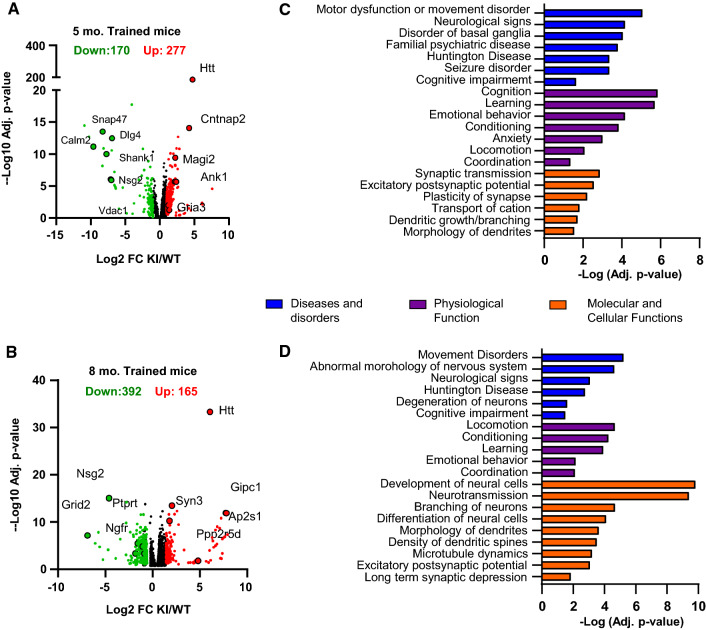
Behavioral training induces differential methylation of synaptic genes in symptomatic *Hdh*^+*/Q111*^ (KI) mice. Volcano plots of the differentially methylated peaks between trained KI and WT animals at 5- (**A**) and 8- (**B**) months of age. Names of representative genes are indicated. IPA of hypomethylated transcripts between trained KI and WT mice at 5- (**C**) and 8- (**D**) months of age. Bar plots showing the -Log (adj. *p* value) of enriched terms belonging to the different categories. Screening threshold: adjusted *p* value < 0.05 and log2 fold change > 1 or < − 1

Our data revealed differential m6A methylation patterns between WT and *Hdh*^+*/Q111*^ mice along the disease progression. Next, we sought to investigate whether mutant huntingtin expression was associated with changes in methylation dynamics in hippocampal cognition. To this aim, we analyzed the differential methylated peaks between *Hdh*^+*/Q111*^ and WT mice after the spatial training in the OLT. We found 277 hypermethylated m6A peaks and 170 hypomethylated m6A peaks at 5 months of age in trained *Hdh*^+*/Q111*^ mice compared to WT mice, while at 8 months only 165 peaks were hypermethylated whereas 392 peaks were hypomethylated (Fig. 3a, b and Supplementary Tables 10 and 11). To deepen about the functional consequences of these changes, IPA pathway analysis between trained *Hdh*^+*/Q111*^ and WT mice was performed (Supplementary Tables 12 and 13 for complete lists of all biological functions and their associated genes), analyzing hypo- and hypermethylated transcripts independently. We found that the strongest enrichment in relevant functional terms was obtained in the hypomethylated transcripts, with an important enrichment in “motor or movement disorder,” including “Huntington´s disease” within both ages (Fig. 3c, d and Supplementary Fig. 6a–d). Importantly, at 8 months of age, training in *Hdh*^+*/Q111*^ mice induced the hypomethylation of genes tightly involved in synaptic transmission when compared to WT trained mice (Fig. 3d). No significant terms could be detected in the hypermethylated transcripts at 8 months, and only a few interesting terms were found in hypermethylated transcripts at 5 months (Data shown in supplementary tables 12 and 13).

### Symptomatic ***Hdh***^+***/Q111***^ mice show aberrant m6A demethylation after hippocampal cognitive training.

**Fig. 4:**
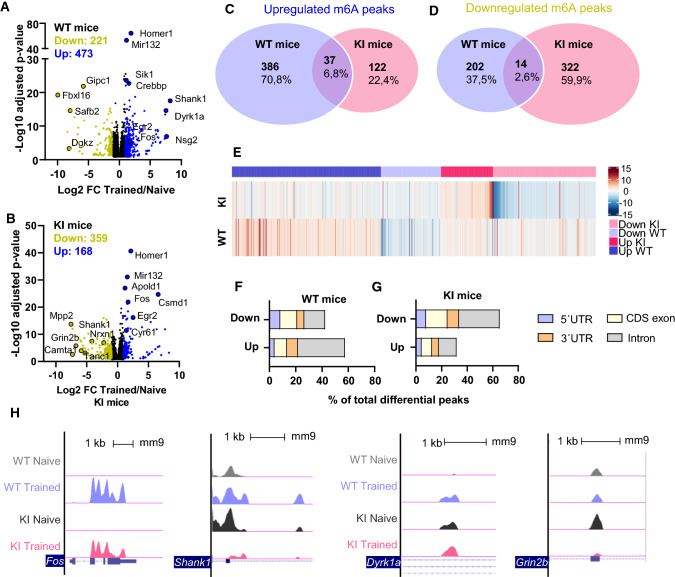
Symptomatic *Hdh*^+ /*Q111*^ (KI) mice exhibit prominent demethylation in response to behavioral training. Volcano plots of the differentially methylated peaks between trained and naive conditions in WT (**A**) and KI **(B**) 8-month-old animals. Names of representative genes are indicated. Venn diagram showing shared and unique differential (**C**) hypermethylated and (**D**) hypomethylated transcripts in WT and KI after training. **E** Heatmap representing the fold change of m6A methylation levels between trained and naive conditions in differentially methylated transcripts identified in WT and KI animals. Distribution analysis of the down- and up-regulated differential m6A peaks between naive and trained conditions in symptomatic disease stages in WT (**F**) and KI (**G**) mice along the four transcript segments: 5′UTR, CDS exon, 3′UTR, intron. Data are shown as percentage of total differential peaks. **H** UCSC Genome browser tracks showing representative differential m6A peaks (The y-axis represents normalized number of reads. Blue thick boxes represent the open reading frame while the blue line represents the untranslated regions (arrows point to the direction of transcription). Screening threshold: adjusted *p* value < 0.05 and log2 fold change > 1 or < − 1

Our previous data have revealed altered m6A RNA patterns between WT and *Hdh*^+*/Q111*^ mice at pre- and symptomatic stages, both in basal and after cognitive engagement conditions. However, when comparing *Hdh*^+*/Q111*^- vs WT-trained mice, we observed a stronger enrichment of differentially methylated genes on the “Synaptic Transmission” function in symptomatic disease stages. To get further insight into the specific changes in m6A associated with cognitive training, we compared the differential m6A peaks in training vs naive conditions within each genotype at 8 months of age (Supplementary Tables 14 and 15). Using the same filtering criteria as above (Fig. 2a, b) we identified in trained vs naive WT mice, 473 hypermethylated m6A peaks and 221 hypomethylated m6A peaks (Fig. 4a). In contrast, when trained *Hdh*^+*/Q111*^ mice were compared to naive *Hdh*^+*/Q111*^ mice, the differential analysis revealed only 168 hypermethylated m6A peaks and 359 hypomethylated m6A peaks (Fig. 4b). The extent of overlap among differential methylation in each genotype at 8 months of age in response to training is shown in Fig. 4c, d. We observed a unique pattern of hypermethylation and hypomethylation in each genotype, with only a minority of differential m6A marked transcripts in common between WT and *Hdh*^+*/Q111*^ mice. In addition, the extent of m6A changes of differential m6A-marked transcripts identified in WT did not resemble the methylation pattern observed in KI (Fig. 4e), re-enforcing the idea that memory-induced m6A methylation is impaired in KI mice. Moreover, the hierarchical heatmap representation of differential methylated transcripts in common (Supplementary Fig. 7a, b) showed a cluster of Immediate-Early Genes (IEGs) which are similarly hypermethylated after the training task (Fig. 4b), in line with previous evidence showing increased methylation of IEGs after behavioral training [39] and a cluster of 34 transcripts with opposite genotype-dependent m6A regulation.

When comparing trained versus naive conditions in both WT and *Hdh*^+*/Q111*^ mice, the percentage of m6A marked transcripts with differential mRNA expression was around 30% (Supplementary Fig. 8a), indicating that m6A might not affect RNA decay or stability in the remaining transcripts but could affect other aspects of RNA function or metabolism. The association between m6A methylation and mRNA expression in the m6A marked transcripts showed a positive and significant correlation between the magnitude of change in expression and the magnitude of m6A peak enrichment in upregulated mRNAs for both genotypes. In contrast, a statistically significant but weak correlation was found between the downregulated mRNA species and m6A peak enrichment, only for *Hdh*^+*/Q111*^ mice (Supplementary Fig. 8b and c). These results suggest that only the addition of m6A to RNA species in response to cognitive engagement correlates with their increased mRNA expression levels in both genotypes. We further analyzed the distribution of the differential m6A peaks along the transcripts. Here, *Hdh*^+*/Q111*^ and WT mice show an equal distribution in CDS exon, 5’UTR and 3′UTR, with the most frequently occurring position being within introns (Fig. 4f and g). Nevertheless, in trained WT mice we observed a higher percentage of upregulated m6A peaks in introns compared with trained *Hdh*^+*/Q111*^ mice, which in turn showed a higher percentage of downregulated m6A peaks (Supplementary Fig. 8d). To illustrate these results, four representative genes showing the m6A methylation pattern have been depicted (Fig. 4h).

**Fig. 5:**
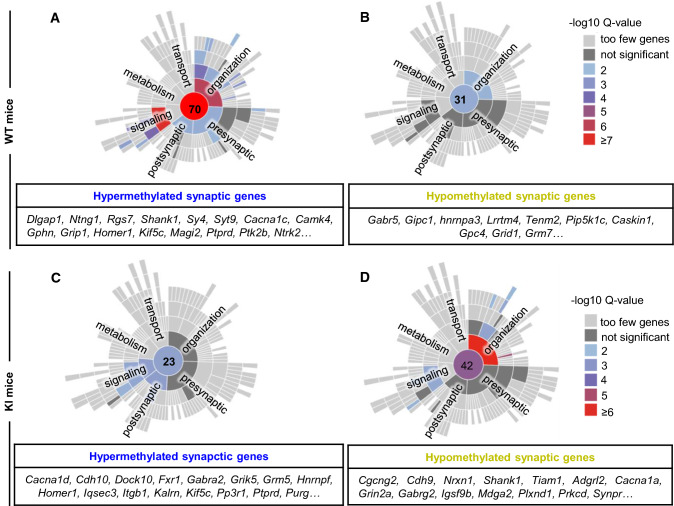
Functional analyses of synaptic genes and gene enrichment of differentially methylated transcripts in trained symptomatic *Hdh*^+*/Q111*^ (KI) mice. Sunburst plots for synaptic GO of differentially hypermethylated (**A**, **C**) and hypomethylated (**B**, **D**) genes in response to the OLT training in 8-month-old WT mice (**A**, **B**) and KI mice (**C**, **D**). In response to the behavioral training task, WT mice show an increased number of hypermethylated genes enriched in synaptic terms, whereas in KI mice genes enriched in synaptic terms are hypomethylated. Numbers in the center indicate number of genes enriched in synaptic terms. Tables below indicate most relevant synaptic genes. GO analysis was performed with the SynGo knowledgebase. Screening threshold: adjusted *p* value < 0.05 and log2 fold change > 1 or < − 1. *GO* gene ontology

Given the role of m6A methylation in synaptic signaling and potentiation during neuronal activation, we next explored the functional signature displayed by the differentially methylated transcripts using the Synaptic Gene Ontologies (SynGO) database. We observed that training-induced hypermethylated genes in WT mice were significantly enriched in “synaptic organization,” “synaptic signaling” and “presynaptic and postsynaptic processes” (Fig. 5a), when compared to enriched hypermethylated genes in *Hdh*^+*/Q111*^ mice (Fig. 5c). By contrast, in *Hdh*^+*/Q111*^ mice, synaptic processes involving “organization” and “synaptic signaling” were enriched for hypomethylated genes (Fig. 5b, d). A similar gene ontology signature of hypomethylation after behavioral training was observed in *Hdh*^+*/Q111*^mice at the age of 5 months, further supporting the idea that m6A methylation is altered during neuronal activity in *Hdh*^+*/Q111*^ mice (Supplementary Fig. 9a–f and Supplementary Tables 16 and 17). These results indicate that the HD mutation impacts on the regulation of m6A methylation, decreasing in synaptic mRNAs after cognitive engagement, which might critically contribute to synaptic and memory dysfunction in *Hdh*^+*/Q111*^ mice.

**Fig. 6:**
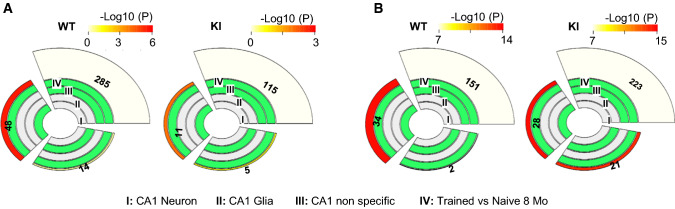
Differential methylated transcripts in *Hdh*^+/*Q111*^(KI) mice in response to training are enriched in CA1 neurons. Multi-layer pie charts showing integration of hypermethylated transcripts (**A**) and hypomethylated transcripts (**B**) from 8-month-old WT and KI mice subjected to behavioral training with cell-type-specific CA1 hippocampal transcriptomic database. Outer levels indicate degree of correlation between the levels marked in green (indicated as − Log10 (*p* value)). The size of each item represents its contribution (number of m6A-modified transcripts) to the inner parent category. Screening threshold: adjusted *p* value < 0.05 and log2 fold change > 1 or < − 1

Finally, since the acquisition of spatial memories during the cognitive training task is known to be modulated by activity-dependent modifications of *cornu ammonis* 1 (CA1) synapses [73], we performed data integration of MeRIP-seq data from 8-month-old WT and *Hdh*^+*/Q111*^ mice after training with data from a cell type-specific CA1 hippocampal transcriptomic database [74]. This analysis showed that at 8 months, transcripts enriched in CA1 neurons, which present structural and functional synaptic deficits in HD [11–13, 70], were highly and significantly overlapping with training-induced hypermethylated transcripts in WT mice, while a lower enrichment was found in *Hdh*^+*/Q111*^ mice hippocampus (48 genes enriched in CA1 neurons in WT mice and 11genes in *Hdh*^+*/Q111*^ mice) (Fig. 6a). In addition, hypomethylated transcripts were also associated with CA1 neuronal-enriched transcripts in WT hippocampus while *Hdh*^+*/Q111*^ hypomethylated transcripts were enriched in both CA1 neuronal and glial enriched transcripts after training (Fig. 6b). Together, these results indicate that m6A dysregulation affects CA1 neuronal transcripts in *Hdh*^+*/Q111*^ mice suggesting a role of m6A in transcriptional remodeling underlying acquisition of spatial memory.

### Subcellular expression of FTO is modulated in WT but not ***Hdh***^+***/Q111***^ mice following a hippocampal cognitive training task

**Fig. 7:**
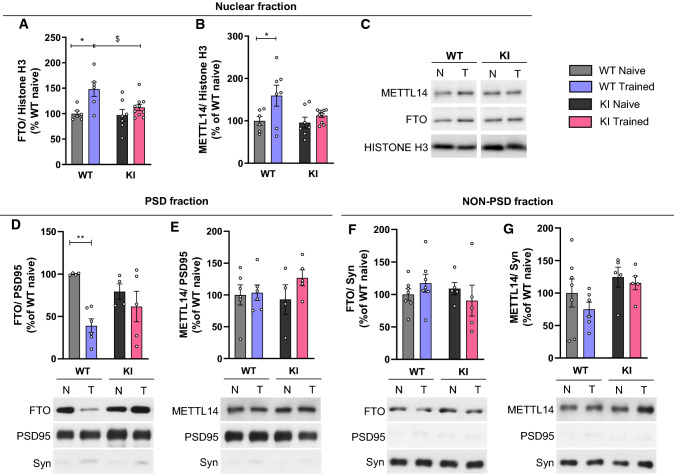
Nuclear and post-synaptic levels of the m6A modifying proteins do not change upon training in symptomatic *Hdh*^+*/Q111*^ (KI) mice. Western Blots for METTL14 and FTO in **A**–**C** nuclear enriched fractions, **D**, **E** post-synaptic (PSD) and **F**, **G** non-PSD fractions from hippocampus of 8-month-old mice subjected to the training in the OLT. **A**–**C** Nuclear fraction was normalized to Histone H3. Two-way ANOVA reported a significant training effect in FTO levels (*F*(1,25) = 10.96, *p* = 0.0028) and in METTL14 levels (*F*(1,26) = 7.101, *p* = 0.0131) (*n* = 6–10 animals/condition). Right: Representative immunoblots **D**, **E** PSD fraction was normalized to PSD95. Two-way ANOVA reported a significant training effect in FTO levels (*F*(1,15) = 11.21, *p* = 0.0044) (*n* = 4–6 animals/condition). Below the graphs: Representative immunoblots (**F**, **G**). Non-PSD fraction was normalized to synaptophysin. (*n* = 5–7 animals/condition). Below the graphs: Representative immunoblots. Data were analyzed using Tukey as a post hoc test. *p < 0.05 and **p < 0.01 compared with WT naive mice. ^$^*p* < 0.05 compared with WT-trained mice. Data are presented as mean ± SEM. *N* naïve, *T* trained, *Syn* synaptophysin

Regulation of m6A in response to sensory experience and learning is supported by methyltransferases and demethylases [28, 35, 75, 76]. To define whether altered m6A methylation patterns in *Hdh*^+*/Q111*^ mice were related with changes in the levels of m6A-modifying proteins, expression of METTL14 and FTO was analyzed in WT and *Hdh*^+*/Q111*^ mice hippocampus under naive and training conditions. Since both enzymes are enriched in the nucleus [25, 27], subcellular fractionation assays in hippocampal lysates were performed to determine the levels of nuclear METTL14 and FTO. At 5 months of age, similar levels of FTO and METTL14 were found in nuclear fractions between genotypes and conditions (Supplementary Fig. 10a–c). By contrast, at 8 months of age a significant increase of both proteins was observed in WT mice in response to hippocampal training while no significant changes were detected in *Hdh*^+*/Q111*^ mice (Fig. 7a–c). Since FTO and METTL14 have also been found in synapsis and distal neuronal processes [36, 40] and given the changes detected at 8 but not 5 months of age following training, we next analyzed the levels of both m6A regulatory enzymes in postsynaptic density (PSD) and non-PSD fractions (Fig. 7d–g). We observed that FTO levels decrease in the postsynaptic fraction in WT mice after training while no changes were observed in *Hdh*^+*/Q111*^ mice (Fig. 7d, e). Altogether, these findings indicate that at a symptomatic stage characterized by hippocampal cognitive deficits, *Hdh*^+*/Q111*^ mice display altered protein levels of FTO and METTL14.

### Inhibition of m6A demethylation in hippocampal CA1 region improves exploratory behavior and spatial memory in ***Hdh***^+***/Q111***^ mice

**Fig. 8:**
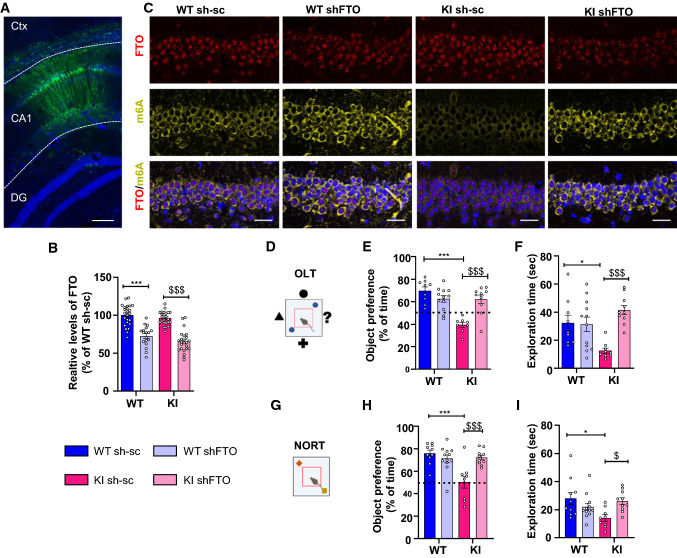
Targeted knockdown of FTO in the dorsal hippocampus of *Hdh*^+*/Q111*^ (KI) mice rescues spatial memory deficits at 8 months of age. **A** Representative image showing AAV injection site in the CA1. Immunological staining shows GFP (green) and DAPI (blue). Scale bar 150 µm. **B** Relative levels of nuclear FTO (Integrated Optical Density) in the CA1 of injected mice. Two-way ANOVA reported a significant treatment effect (shFTO) (*F*(1,128) = 92.73, *p* < 0.0001) (*n* = 3–4 animals/condition) (3 slices/animal were analyzed, 2 images for each slice). **C** Representative confocal images of dorsal CA1 transduced with sh-sc or shFTO in WT and KI mice. Triple staining showing FTO (red), m^6^A (yellow) and DAPI (blue). Scale bar 5 µm. Schematic illustration of OLT (**D**) and NORT (**G**) behavioral assessment. **E** Percentage of time exploring the displaced object for WT sh-sc, WT shFTO, KI sh-sc and KI shFTO. Two-way ANOVA reported a significant interaction effect (*F*(1,35) = 21.00, *p* < 0.0001), a significant treatment effect (shRNA) (*F*(1,35) = 5.819, *p* = 0.0212) and a significant genotype effect (*F*(1, 35) = 22.55, *p* < 0.0001). **F** Exploration time (sec) during the testing session of OLT. Two-way ANOVA reported a significant interaction effect (*F*(1, 36) = 11.63, *p* = 0.0016) and a significant treatment effect (shRNA) (F(1, 36) = 10.09, p = 0.0031. **H** Percentage of time exploring the new object for WT sh-sc, WT shFTO, KI sh-sc and KI shFTO. Two-way ANOVA reported a significant interaction effect (*F*(1,37) = 14.08, *p* = 0.0006), a significant treatment effect (shRNA) (*F*(1, 37) = 6.154, *p* = 0.0178) and a significant genotype effect (*F*(1,37) = 12.22, *p* = 0.0012). **I** Exploration time (sec) during the testing session of NORT for WT sh-sc, WT shFTO, KI sh-sc and KI shFTO. Two-way ANOVA reported a significant interaction effect (*F*(1,37) = 8.840, *p* = 0.0052). Dashed line shows chance levels for exploration. Two-way ANOVA with Tukey’s multiple comparisons test; *p < 0.05 and ***p < 0.001 compared with WT sh-sc mice; ^$^*p* < 0.05 and ^$$$^*p* < 0.001 compared with KI sh-sc mice. Data are presented as mean ± SEM (*n* = 9–12 per genotype). *DG* dentate gyrus

In view of the results showing decreased levels of m6A methylation in synaptic-related genes in trained *Hdh*^+*/Q111*^ mice, we hypothesized that synaptic and cognitive defects in HD mice could be related with a dysregulation of m6A modification dynamics. To test our hypothesis, we investigated the effect of FTO knockdown on the hippocampal-dependent memory deficits previously described in *Hdh*^+*/Q111*^ mice [13]. Adeno-associated viruses (AAV) encoding eGFP scramble control shRNA or shFTO were injected bilaterally into the dorsal hippocampus of WT and *Hdh*^+*/Q111*^ mice at 8 months (Fig. 8a). To test the efficiency of shRNA in knocking down FTO expression, we measured by immunofluorescence intensity the levels of FTO levels in the CA1 region following shFTO injection. A significant reduction of around 30% was found when compared to scramble control (Fig. 8b). Moreover, immunofluorescence co-labelling studies of FTO and m6A confirmed that m6A immunoreactivity was reduced in the pyramidal neurons of the CA1 in *Hdh*^+*/Q111*^ mice compared with WT mice while increased in *Hdh*^+*/Q111*^ mice following shFTO injection (Fig. 8c). To study the effect of FTO knockdown in the hippocampus, we first analyzed spatial memory by the OLT (Fig. 8d) [77, 78]. During training, all mice similarly explored both objects, indicating no object or place preferences between genotypes (Supplementary Fig. 11a and b). However, *Hdh*^+*/Q111*^ mice displayed decreased total exploration time (Supplementary Fig. 11c). When long-term spatial memory was assessed 24 h after training, *Hdh*^+*/Q111*^ mice injected with AAV control shRNA exhibited a significantly lower preference for the object displaced to the new location when compared to WT mice and *Hdh*^+*/Q111*^ mice injected with AAV shFTO (Fig. 8e), confirming previous results from our lab showing hippocampal-dependent deficits in *Hdh*^+*/Q111*^ mice [13, 16]. Importantly, FTO knockdown in *Hdh*^+*/Q111*^ mice lead not only to a significant preference for the new object location but also to an increase in total exploration time indicating that recovery of m6A levels in *Hdh*^+*/Q111*^ mice rescue spatial memory deficits (Fig. 8e, f). Similar results were obtained when we evaluated recognition memory by Novel Object Recognition Test (NORT) (Fig. 8g) [79]. No significant object preference between genotypes was found during the training period, while a significant decrease in total exploration time was observed in *Hdh*^+*/Q111*^ compared to WT mice (Supplementary Fig. 11d–f). Twenty-four hours after training, *Hdh*^+*/Q111*^ mice injected with AAV control shRNA exhibited a significantly lower preference for the novel object compared with WT mice and with *Hdh*^+*/Q111*^ mice injected with AAV shFTO (Fig. 8h), indicating preserved long-term recognition memory in *Hdh*^+*/Q111*^ mice with normalized levels of FTO and restored levels of m6A. As previously observed during OLT test, memory rescue was accompanied by an increase in exploratory behavior (Fig. 8i). Taken together, these data show that modulation of m6A levels in symptomatic *Hdh*^+*/Q111*^ mice by knockdown of FTO expression in the CA1 of the dorsal hippocampus restores HD spatial and recognition memory.

## Discussion

Our study provides for the first time, evidence of m6A hypermethylation in relevant HD and synaptic-related genes in the hippocampal transcriptome of *Hdh*^+*/Q111*^ mice. We also found that m6A is aberrantly regulated in an experience-dependent manner in the HD hippocampus leading to demethylation of important components of synapse organization which could underlie HD cognitive deficits. In agreement with these data, we show that FTO knockdown in HD mice improves hippocampal spatial and recognition memories. Overall, these findings suggest that m6A RNA modifications represent a new RNA post-transcriptional signature underlying gene expression dysregulation and cognitive deficits in HD.

Here, we identified in a HD mice model, hypermethylation in several transcripts of genes previously described to be altered in HD such as, *Pde10a* [80, 81], *Eif3b* [82], *Kalrn* [47], *Ntrk2* [83], *Grin2b* [84, 85], *Dyrk1a* [86] and *Htt* itself. These findings suggest a possible contribution of m6A modifications in the HD physiopathology. To date studies in Alzheimer´s and Parkinson´s Disease have suggested that m6A dysregulation is a common feature of many neurodegenerative diseases [42, 46] and have emphasized the importance of an appropriate m6A equilibrium for an accurate hippocampal function. Accordingly, we have shown that hypermethylated transcripts in symptomatic *Hdh*^+*/Q111*^ mice under naive conditions are enriched in synapse-related functions, supporting a role of m6A in mediating HD synaptic and memory deficits. This finding reveals a new layer of dysregulation in the expression of synaptic genes, a process previously associated in HD to changes in the activity or levels of transcription factors and epigenetic modifications [14–16, 87, 88]. Indeed, recent studies have revealed that m6A is present in the synaptic transcriptome, modifying in a selective manner different transcripts and shaping the synaptic proteome [40], which support our hypothesis that disruption or dysregulation of m6A modifications would impact memory processes in HD mice. In this regard, behavioral studies have demonstrated the activity-dependent nature of neuronal m6A in response to behavioral experience and memory formation in different brain regions [35, 36, 76]. In accordance, we found that mRNA methylation was actively regulated in the dorsal hippocampus of both WT and *Hdh*^+*/Q111*^ mice in response to spatial learning, though a different m6A pattern was observed in the transcriptome of *Hdh*^+*/Q111*^ mice when compared to WT mice. Thus, although both genotypes showed similar methylation in IEGs and synaptic genes known to be target of m6A modifications [39, 76, 89, 90], *Hdh*^+*/Q111*^ mice failed to increase methylation or induced demethylation of some synaptic genes critical for proper learning. Since an increase of m6A in the mouse hippocampus or prefrontal cortex in response to learning has been associated with the constrain of the sorting efficiencies or turnover of nascent RNAs, the reduced m6A methylation observed in *Hdh*^+*/Q111*^ hippocampus could indicate that the degradation and stability of several synaptic genes is affected in these mice.

An interesting finding of this study is the consistent and differentially methylated peak in intron 1 of m*Htt,* in both 5- and 8-month-old *Hdh*^+*/Q111*^ naive and trained mice. Remarkably, studies have shown that the increased number of CAG repeats in *mHtt* leads to a slower transcription rate of RNA polymerase II in this intronic region [91]. In turn, slow transcription speeds have been associated to an elevated m6A content [92] pointing to a possible link between the number of CAG repeats and m6A deposition in Htt intron. This evidence further strengthens the contribution of m6A in HD physiopathology, although its function on Htt RNA fate remains to be investigated.

RNA m6A methylation can have mixed effects beyond regulation of transcript abundance. Indeed, the specific location of m6A can determine alternative polyadenylation usage [65, 93], deadenylation-mediated RNA degradation [64], and alternative splicing [34, 67]. In this sense, when comparing naive WT and *Hdh*^+*/Q111*^ mice, we observed a differential m6A methylation in the distinct transcript regions (5´ and 3´UTR, exons and introns), which could induce such effects on downstream mRNA processing and metabolism in the HD condition. Supporting this possibility, we found several transcripts hypermethylated that have previously been described to be affected by mis-splicing or polyA changes [18, 21]. Majority of the m6A peaks of the methylated transcripts that overlap with mis-spliced genes are found in introns and exons, which are more likely to undergo alternative splicing as previously described [29]. Indeed, m6A modifications in introns are associated with long, slowly processed introns and alternative splicing events [67].

Our data also suggest a possible role of m6A in the activity-dependent alternative splicing processes required to achieve homeostatic control of neuronal output in health and diseased states. We observed under training conditions that most upregulated or downregulated peaks in WT and *Hdh*^+*/Q111*^ mice occur in introns. In the mammalian nervous system, alternative pre-mRNA splicing generates distinct functional isoforms that play key roles in normal physiology, supporting development, plasticity, complex behaviors, and cognition [94]. Strikingly, among the differential methylated genes in *Hdh*^+*/Q111*^ mice after training we identified *Shank1* and *Nrxn1*. Shank 1, which plays an important role as scaffold protein in the formation of the PSD, contains diverse domains that appears to be regulated by alternative splicing [95]. Neurexin 1 (*Nrxn1*) also generates isoforms by alternative splicing enhancing postsynaptic NMDA-receptor-mediated synaptic responses [96]. All this evidence supports our hypothesis that m6A dysregulation could affect the fine tune of alternative splicing in *Hdh*^+*/Q111*^ mice, both under naive and trained conditions.

To understand the differential dynamic nature of the m6A RNA modifications observed in *Hdh*^+*/Q111*^ mice, we investigated the protein expression levels of FTO and METTL14 at different disease stages. In accordance with evidence showing correlation between abundance of METTL3 and METTL14 and learning efficiency in mouse hippocampus [39], we found increased nuclear METTL14 levels in WT mice after training. Importantly, this nuclear increase in the levels of METTL14 in response to neuronal activity demands during learning was not observed in *Hdh*^+*/Q111*^ mice and could be associated to the reduced methylation of transcripts observed after training. On the other hand, our data show that in WT mice after a memory-inducing activity, FTO levels rapidly increase in the nucleus and decrease in postsynaptic fractions. Within the nucleus, the observed increase of FTO levels in WT mice could clearly underlie synaptic remodeling by RNA processing as previous studies reported that overexpression of FTO results in increased co-localization with RNA processing factors [97]. On the contrary, memory-inducing activity is also known to rapidly decrease FTO in synapses increasing m6A to facilitate translation [35, 36, 76]. These local changes in FTO that are observed in response to behavioral experiences are also compatible with a role in regulation of synaptic plasticity in memory functions [75]. Remarkably, in *Hdh*^+*/Q111*^ mice FTO levels remain unchanged and steady FTO levels likely maintain a demethylation activity that would inhibit or disrupt translation or proper processing of synaptic genes which have already been described to be altered in HD [98–100].

Consistent with this dysregulation of FTO levels in *Hdh*^+*/Q111*^ mice, we detected an increased demethylation pattern in critical genes involved in memory processes and enriched in CA1 neurons. In accordance, FTO knockdown in CA1 region before training was sufficient to enhance spatial and recognition memory in *Hdh*^+*/Q111*^ mice. Interestingly, FTO knockdown improved the exploratory behavior in *Hdh*^+*/Q111*^ mice raising the question whether the enhanced memory observed by FTO knockdown was due to this increased exploratory behavior. It is known that processes like exploration, novelty and choice affects hippocampal function and memory via dopaminergic signaling which can be modulated by FTO [101–105]. Alterations in hippocampal dopaminergic signaling in HD mice has been reported to participate in hippocampal-dependent learning and memory deficits [106]. Hence, we hypothesize that FTO knockdown in CA1 neurons increased methylation of genes involved in the control of dopamine circuitry neurotransmission during training, promoting exploratory behavior and facilitating learning. Further analysis of FTO target genes in HD mice would provide better understanding of the signaling pathways affected by FTO dysregulation during memory formation in the affected HD brain.

The m6A dysregulation demonstrated in this study provides a missing signature that might define the aberrant post-transcriptional events underlying neuronal dysfunction in HD. Furthermore, our results open new avenues of investigation into the pathophysiological function of m6A during disease progression which might guide the development of new therapeutic strategies for HD.

### Supplementary Information

Below is the link to the electronic supplementary material.

Supplementary file1 (PDF 1445 KB)

Supplementary file2 (XLSX 237 KB)

## Data Availability

The sequence data, narrow Peak, and alignment data supporting the conclusions of this article are available in the NCBI GEO repository under the accession code GSE175618.

## Declarations

### Study approval

All procedures were performed in compliance with the National Institutes of Health Guide for the Care and Use of Laboratory Animals and approved by the local animal care committee of the Universitat de Barcelona (448/17) and the Generalitat de Catalunya (9878 P2), in accordance with the European (2010/63/EU) and Spanish (RD53/2013) guidelines for the care and use of laboratory animals.
