# Supplementary material for: Altered m6A RNA methylation contributes to hippocampal memory deficits in Huntington’s disease mice
Source: Cell Mol Life Sci. 2022 Jul 11;79(8):416. doi: 10.1007/s00018-022-04444-6 (PMC9276730; doi:10.1007/s00018-022-04444-6)
Supplement: Supplementary file 1 — Supplementary file1 (PDF 1445 KB) [file 18_2022_4444_MOESM1_ESM.pdf]

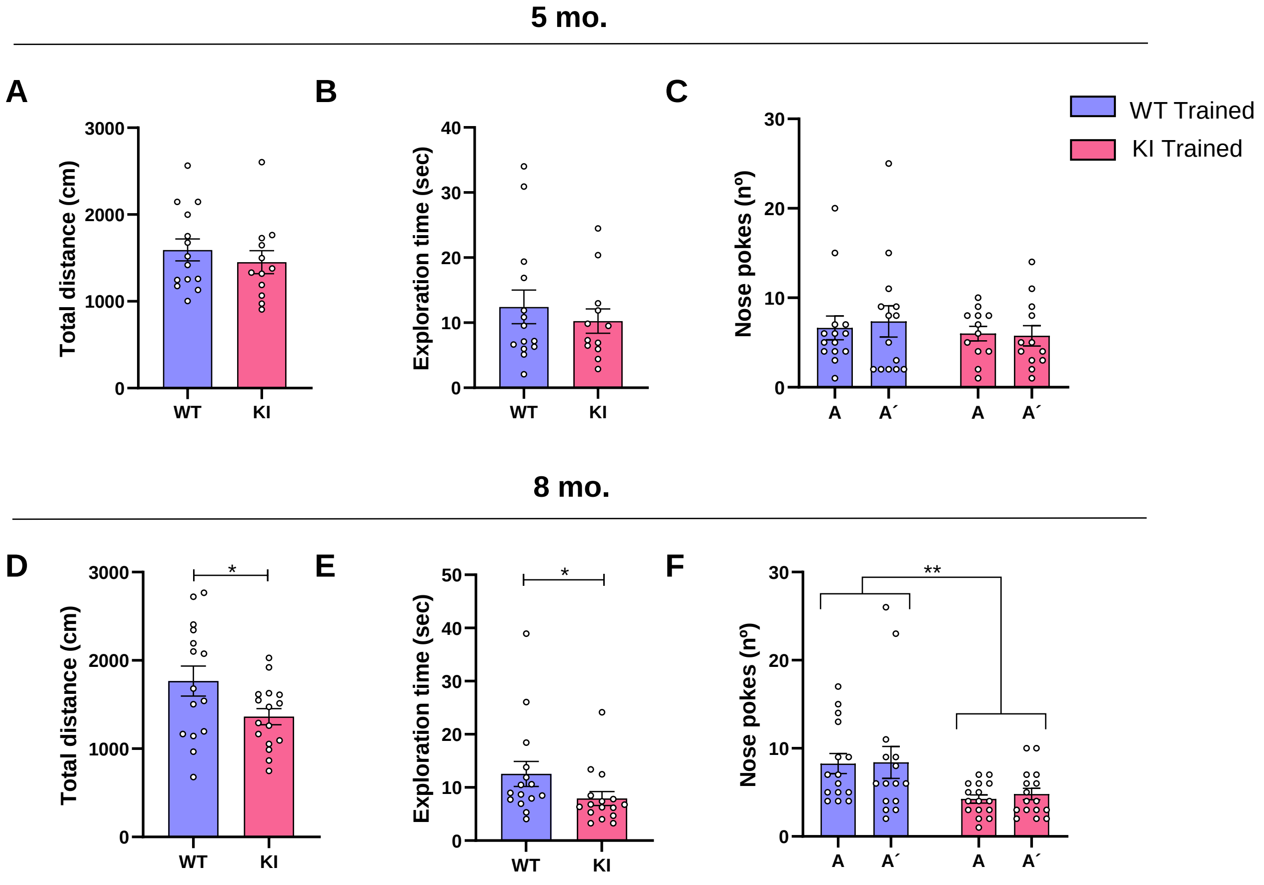

Supplementary Figure 1. Behavior analysis of WT and Hdh$^{+/Q111}$ (KI) mice during the training on the OLT. (**A-C**) Total distance traveled (cm) in the open field arena (**A**), exploration time of the objects (sec) (**B**) and number of nose pokes to objects A and A' (**C**) during the training of 10 minutes on the OLT for WT and KI mice at 5 months of age. (**D-F**) Total distance traveled (cm) in the open field arena (**D**), exploration time of the objects (sec) (**E**) and number of nose pokes to objects A and A' (**F**) during the 10 minutes of training on the OLT for WT and KI mice at 8 months of age. No object preference could be detected. Significant differences were found between genotypes at 8 months of age in the total distance travelled (Student's two-tailed t-test, p=0.0426), the exploration time (Mann-Whitney test, p=0,0157) and the number of nose pokes to the objects (Two-way ANOVA reported a significant genotype effect (F(1,58)=11.59, p=0.0012)). Data represent the mean ± SEM (5 months: n=12-14 per genotype; 8 months: n=15-16 per genotype).

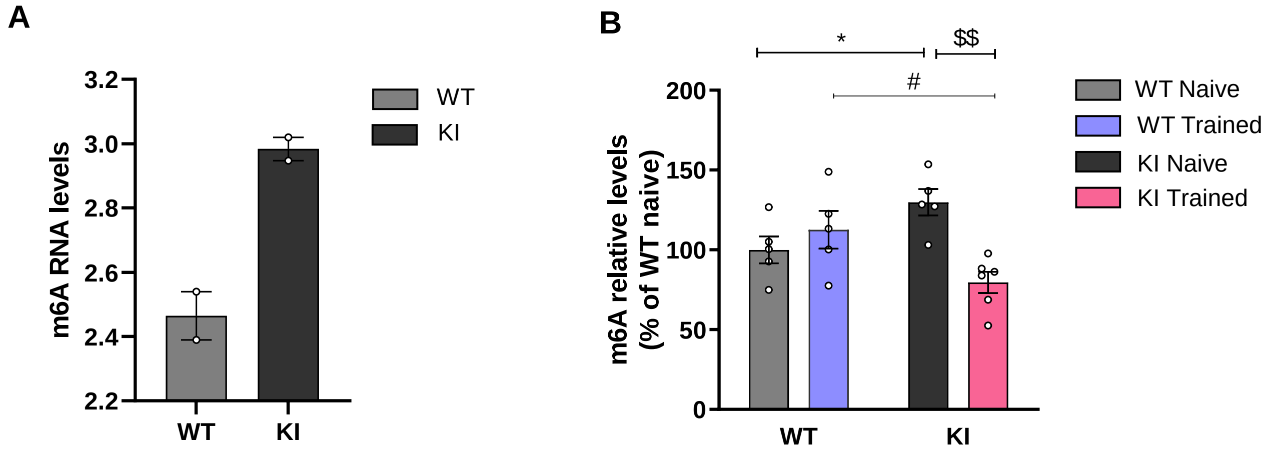

Supplementary Figure 2. m6A RNA modification levels in hippocampal RNA derived from 8 months old mice. (**A**) Histogram depicts the hippocampal levels of m6A in total RNA measured via LC-MS/MS in 8 months old mice under basal conditions (no cognitive training). m6A RNA levels: n/10^4 rNS (number of modified ribonucleosides per 10^4 unmodified ribonucleosides). Specific measurement of only m6A (n = 2 animals/genotype). (**B**) Global m6A assay on total hippocampal RNA from naive and trained WT and KI mice (n=5-6/condition). KI mice show a significant decrease in m6A levels after the OLT training task. Two-way ANOVA with Tukey's multiple comparisons test; Two-way ANOVA reported a significant interaction effect ($F(1,17)=12.69$, $p=0.0024$) and a significant training effect ($F(1,17)=4.574$, $p=0.0473$); $^{*}P < 0.05$ compared with WT naive mice. $^{\$\$}P < 0.01$ compared with KI naive mice. $^{\#}P < 0.05$ compared with WT trained mice. Data are presented as mean ± SEM.

Supplementary Figure 2

A

KI vs WT Naive	5 mo	8 mo
DE mRNA with m6A changes	201	123
DE mRNA	529	327
DMET genes	419	526
% of m6A tagged genes with RNA changes	47	23
% of RNA species with m6A changes	38	37

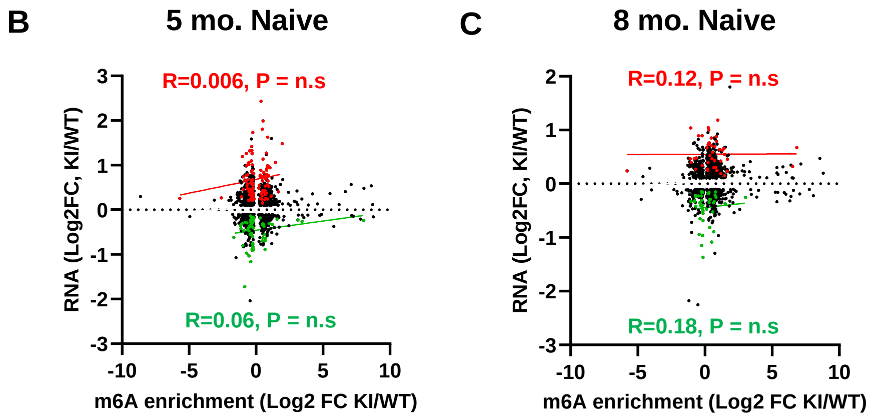

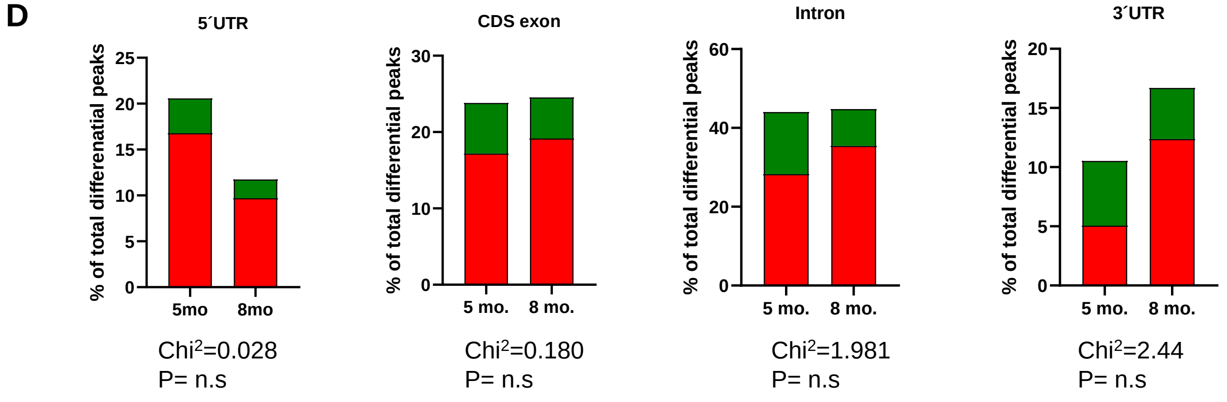

Supplementary Figure 3. Distribution analysis of m6A peaks and correlation analysis between mRNA expression levels and m6A peak enrichment in naive mice. (**A**) Table showing comparison of differential mRNA expression with differential m6A peaks in 5- and 8- months old naive mice (KI vs WT). (**B,C**) Correlation between the FC of mRNA abundance in KI mice relative to WT mice and the log2 ratio of m6A enrichment in naive 5- (**B**) and 8- (**C**) months old mice. Green, red and black dots represent downregulated peaks in KI mice, upregulated peaks, and non-differential peaks, respectively. P values were calculated from a Pearson´s product-moment correlation. No significant correlation between mRNA expression and m6A peaks could be detected. (**D**) Differential distribution analysis of the down- and up-regulated m6A peaks (KI vs WT) between 5- and 8- months old mice in four transcript segments: 5'UTR, CDS exon, 3'UTR, intron. Data is shown as percentage of total differential peaks. Green represents downregulated peaks and red represents upregulated peaks in KI mice. Statistical analysis was performed using Chi^2. All the differential m6A peaks show similar distribution at 5- and 8- months of age. DE, differential expression; DMET, differentially methylated genes.

Supplementary Figure 3

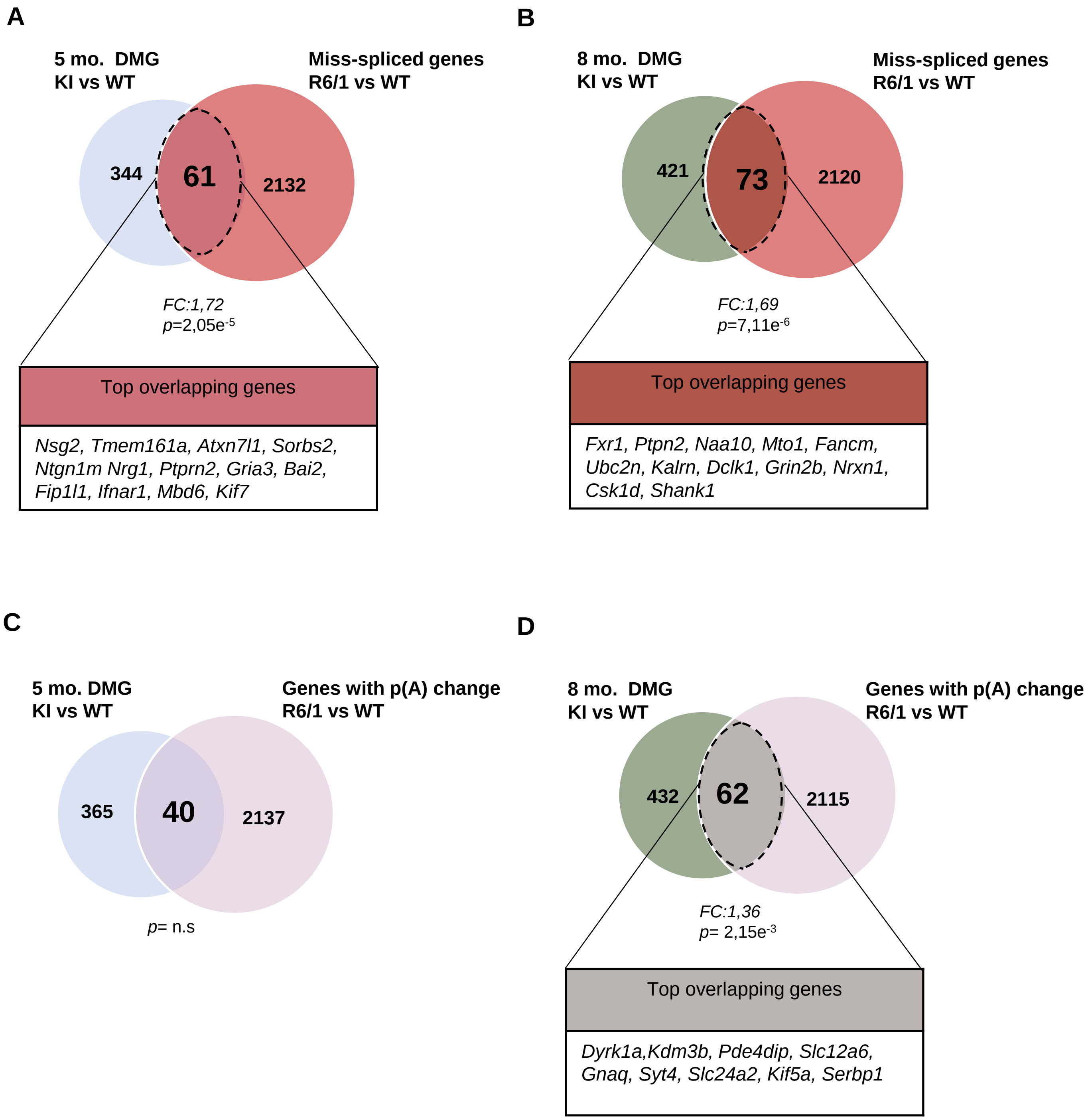

Supplementary Figure 4. Differential m6A peaks are enriched in genes described to be aberrantly processed in HD. (**A,B**) Venn diagram of the overlap of differential m6A marked genes between WT and KI mice at 5- (**A**) and 8- (**B**) months of age with mis-spliced genes in the striatum of 3,5 months old R6/1 HD mice (RNAseq data from Elorza et al., 2021 (18). Tables below diagrams indicate top overlapping genes (genes with highest FC in m6A levels). (**C,D**) Venn diagram of the overlap of differential m6A marked genes between WT and KI mice at 5- (**C**) and 8- (**D**) months of age with genes with altered poly(A) tail length in the striatum of 7-8 months old R6/1 HD mice (RNAseq data from Picó S et al 2021 (21)). Table below diagram indicates top overlapping genes at 8 months of age (genes with highest FC in m6A levels). Correlation analysis was performed with the hypergeometric p-value calculator of the Graeber Lab. FC and p-values are indicated below each Venn diagram. DMG: Differential methylated genes

Supplementary Figure 4

A

Top diseases and Bio Functions 5 mo. naive hypomethylated

Diseases and disorders	p-value range	# molecules
Cancer	2.69E-02 - 2.14E-06	12
Organismal injury and abnormalities	3.49E-02 - 2.14E-06	55
Reproductive system disease	2.69E-02 - 2.14E-06	12
Immunological disease	3.25E-02 - 1.19E-04	14
Inflammatory disease	3.29E-02 - 1.19E-04	14
**Molecular and cellular functions**		
Cell to cell signaling and interaction	3.57E-02 - 8.95E-06	26
Cellular function and maintenance	3.59E-02 - 1.74E-04	28
Cellular growth and proliferation	3.59E-02 - 2.89E-04	24
Cell morphology	3.60E-02 -3.50E-04	27
Cell death and survival	3.22E-02 -8.02E-04	21
**Physiological system development and function**		
Nervous system development and function	3.36E-02 - 8.95E-06	21
Behavior	3.22E-02 - 1.74E-04	10
Hematological system development and function	3.59E-02 - 1.74E-04	15
Embryonic development	3.59E-02 - 2.89E-04	24
Organ development	3.59E-02 - 2.89E-04	24

B

Top diseases and Bio Functions 5 mo. naive hypermethylated

Diseases and disorders	p-value range	# molecules
Developmental disorder	1.43E-02 - 2.43E-06	42
Organismal injury and abnormalities	1.56E-02 - 2.43E-06	94
Neurological disease	1.56E-02 - 1.19E-05	55
Connective tissue disorders	1.48E-02 - 1.20E-05	28
Skeletal and muscular disorders	1.48E-02 - 1.20E-05	30
**Molecular and cellular functions**		
Cellular assembly and organization	1.48E-02 - 2.88E-05	41
Cellular function and maintenance	1.48E-02 - 2.88E-05	47
Cell morphology	1.56E-02 - 4.14E-05	43
Cellular growth and proliferation	1.47E-02 - 7.84E-05	53
Cellular development	1.52E-02 - 9.34E-05	63
**Physiological system development and function**		
Embryonic development	1.47E-02 - 1.10E-05	75
Organismal development	1.56E-02 - 1.10E-05	90
Connective tissue development and function	1.52E-02 - 1.20E-05	32
Skeletal and muscular system development and function	1.48E-02 - 1.20E-05	39
Tissue development	1.52E-02 - 1.20E-05	85

C

Top diseases and Bio Functions 8 mo. naive hypomethylated

Diseases and disorders	p-value range	# molecules
Immunological disease	2.50E-02 - 2.59E-04	3
Organismal injury and abnormalities	4.93E-02 - 2.59E-04	39
Cardiovascular disease	3.72E-02 - 6.16E-04	7
Ophtalmic disease	4.93E-02 - 6.16E-04	4
Connective tissue disroders	2.91E-02 – 2.02E-03	3
**Molecular and cellular functions**		
Carbohydrate metabolism	4.57E-02 - 1.45E-05	3
Cell death and survival	4.93E-02 - 1.75E-05	6
Lipid metabolism	3.31E-02 - 1.75E-05	4
Samll molecule biochemistry	4.93E-02 - 1.75E-05	11
Drug metabolism	2.91E-02 – 5.22E-05	3
**Physiological system development and function**		
Tissue morphology	4.93E-02 - 1.73E-04	23
Hematological system development and function	4.93E-02 - 2.59E-04	9
Lymphoid tissue structure and development	4.93E-02 - 2.59E-04	5
Behavior	4.19E-02 - 3.25E-04	13
Cardiovascular system development and function	4.53E-02 - 6.16E-04	10

D

Top diseases and Bio Functions 8 mo. naive hypermethylated

Diseases and disorders	p-value range	# molecules
Neurological disease	1.53E-02 - 9.43E-06	70
Organismal injury and abnormalities	1.53E-02 - 9.43E-06	122
Cardiovascular disease	1.45E-02 - 8.03E-05	32
Developmental disorder	1.53E-02 - 8.03E-05	43
Cancer	1.53E-02 - 1.18E-04	30
**Molecular and cellular functions**		
Cell to cell signalling and interaction	1.53E-02 - 5.00E-10	67
Molecular transport	1.39E-02 - 2.63E-07	58
Cell morphology	1.53E-02 - 4.86E-07	84
Cellular assembly and organization	1.53E-02 - 4.86E-07	78
Cellular function and maintenance	1.53E-02 - 4.86E-07	97
**Physiological system development and function**		
Nervous system development and function	1.53E-02 - 1.58E-09	104
Behavior	1.44E-02 - 3.78E-09	52
Cardiovascular system development and function	1.53E-02 - 8.15E-07	57
Embryonic development	1.53E-02 - 8.15E-07	76
Organ development	1.53E-02 - 8.15E-07	62

Supplementary Figure 5. Ingenuity Pathway Analysis (IPA) of differential hypo- and hypermethylated hippocampal m6A marked transcripts in naive mice. Tables show the top five pathways belonging to the categories “Disease and Disorders”, “Molecular and Cellular functions” and “Physiological System development and function” in 5- (**A**) and 8- (**B**) months old naive mice (KI vs WT comparison). Screening threshold: adjusted p-value <0.05 and log2 fold change > 1 or < -1. These pathways emerged following IPA “Core Analysis.”

Supplementary Figure 5

A

Top diseases and Bio Functions 5 mo. trained hypomethylated

Diseases and disorders	p-value range	# molecules
Neurological disease	3.49E-02 - 1.59E-08	39
Organismal injury and abnormalities	3.51E-02 - 1.59E-08	53
Psychological disorders	2.01E-02 - 4.44E-07	20
Hereditary disorder	2.34E-02 - 1.00E-06	16
Skeletal and muscular disorders	3.49E-02 - 2.13E-06	17
**Molecular and cellular functions**		
Cell to cell signaling and interaction	3.49E-02 - 4.15E-06	25
Small molecule biochemistry	3.49E-02 - 2.32E-05	17
Cellular movement	2.19E-02 - 3.19E-05	6
Cell morphology	3.13E-02 -1.16E-04	28
Molecular transport	3.49E-02 -2.89E-04	20
**Physiological system development and function**		
Behavior	3.49E-02 - 9.05E-10	29
Nervous system development and function	3.49E-02 - 2.29E-06	40
Organ morphology	3.49E-02 - 2.29E-06	27
Organismal development	3.49E-02 - 2.29E-06	36
Auditory and vestibular system development and function	2.91E-02 - 2.33E-05	6

B

Top diseases and Bio Functions 5 mo. trained hypermethylated

Diseases and disorders	p-value range	# molecules
Endocrine system disorders	1.89E-02 - 2.83E-05	13
Organismal injury and abnormalities	2.56E-02 - 2.83E-05	68
Reproductive system disease	1.89E-02 - 2.83E-05	14
Ophthalmic disease	1.72E-02 - 4.26E-05	7
Developmental disorder	2.50E-02 - 2.20E-04	23
**Molecular and cellular functions**		
Cell to cell signaling and interaction	2.01E-02 - 8.72E-09	37
Cellular assembly and organization	2.09E-02 - 8.72E-09	50
Cellular death and survival	2.56E-02 - 1.61E-06	34
Cell development	2.52E-02 - 1.67E-05	51
Cellular growth and proliferation	2.52E-02 - 1.67E-05	45
**Physiological system development and function**		
Nervous system development and function	2.52E-02 - 8.72E-09	67
Tissue morphology	2.56E-02 - 8.72E-09	55
Tissue development	2.52E-02 - 1.67E-05	56
Organ morphology	2.31E-02 - 2.83E-05	33
Reproductive system development and function	2.09E-02 - 2.83E-05	6

C

Top diseases and Bio Functions 8 mo. trained hypomethylated

Diseases and disorders	p-value range	# molecules
Neurological disease	1.37E-02 - 1.40E-08	80
Organismal injury and abnormalities	1.37E-02 - 1.40E-08	126
Hereditary disorder	1.37E-02 - 1.07E-05	26
Psychological disorders	1.37E-02 - 1.25E-05	35
Ophtalmic disease	1.37E-02 – 1.61E-05	14
**Molecular and cellular functions**		
Cellular development	1.37E-02 - 4.80E-14	71
Cell growth and proliferation	1.37E-02 - 4.80E-14	62
Cell to cell signaling and interaction	1.37E-02 - 3.78E-13	68
Cell morphology	1.37E-02 - 1.10E-08	67
Cellular assembly and organization	1.37E-02 – 5.81E-08	64
**Physiological system development and function**		
Nervous system development and function	1.37E-02 - 4.80E-14	112
Tissue development	1.37E-02 - 4.80E-14	88
Organismal development	1.37E-02 - 2.55E-08	105
Behavior	1.31E-02 - 9.63E-08	52
Embryonic development	1.37E-02 - 2.03E-07	56

D

Top diseases and Bio Functions 8 mo. trained hypermethylated

Diseases and disorders	p-value range	# molecules
Neurological disease	3.90E-02 - 1.93E-04	24
Organismal injury and abnormalities	3.90E-02 - 1.93E-04	45
Psychological disorders	3.26E-02 - 2.58E-04	8
Endocrine system disorders	3.26E-02 - 6.40E-06	10
Reproductive system disease	3.45E-02 - 6.40E-05	14
**Molecular and cellular functions**		
Cellular assembly and organization	3.26E-02 - 9.75E-5	20
Cell to cell signalling and interaction	3.90E-02 - 1.30E-04	17
Cell death and survival	3.01E-02 - 1.93E-04	15
Cellular compromise	3.90E-02 - 1.93E-04	13
Cell cycle	3.90E-02 - 2.58E-04	10
**Physiological system development and function**		
Behavior	3.26E-02 - 1.21E-04	14
Nervous system development and function	3.90E-02 - 1.30E-04	31
Tissue morphology	3.57E-02 - 1.93E-04	25
Organ morphology	3.90E-02 - 2.58E-04	22
Organismal development	3.90E-02 - 2.58E-04	30

Supplementary Figure 6. Ingenuity Pathway Analysis (IPA) of the differentially hypo- and hypermethylated hippocampal transcripts in trained mice. Tables show the top five pathways belonging to the categories “Disease and Disorders”, “Molecular and Cellular functions” and “Physiological System development and function” in 5- (**A,B**) and 8- (**C,D**) months old mice subjected to the training on the OLT (KI vs WT comparison). Screening threshold: adjusted p-value <0.05 and log2 fold change > 1 or < -1.

Supplementary Figure 6

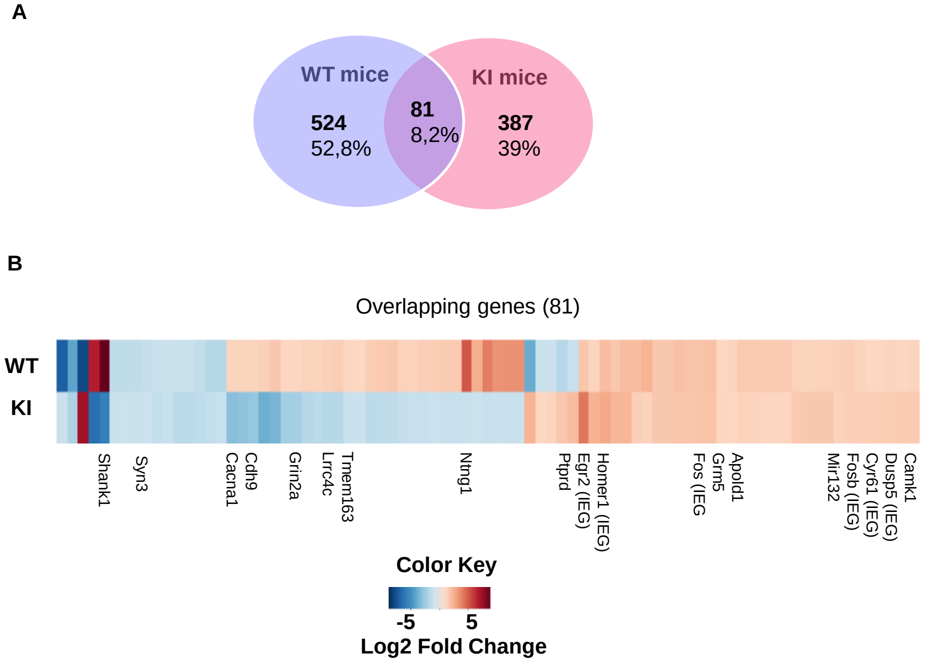

Supplementary Figure 7. Cognitive engagement modulates m6A levels differently in a shared subset of transcripts in symptomatic WT and *Hdh*$^{+/Q111}$ (KI) mice. (A) Venn diagram showing shared and unique differential m6A marked genes between trained and naive conditions in WT and KI 8 months old animals. **(B)** Heatmap representation of the 81 overlapping genes shown in (A). Log2FC of the differentially methylated peaks in response to the training task is represented for each genotype. Names of representative genes are indicated.

Supplementary Figure 7

A

Trained vs Naive	WT	KI
DE mRNA with m6A changes	160	123
DE mRNA	490	453
DMET genes	605	407
% of m6A tagged genes with RNA changes	32	27
% of RNA species with m6A changes	26	30

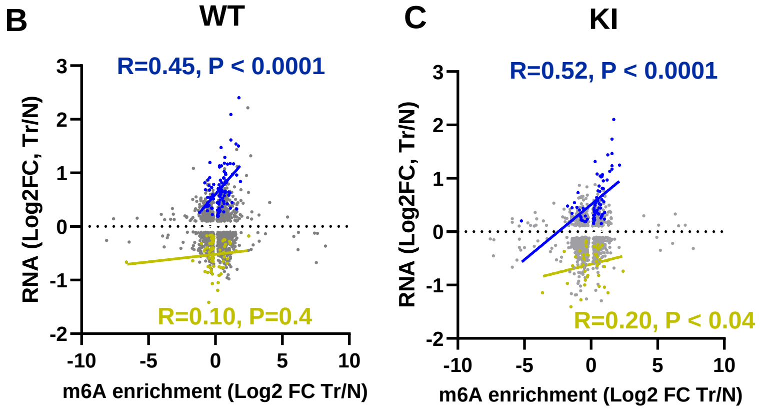

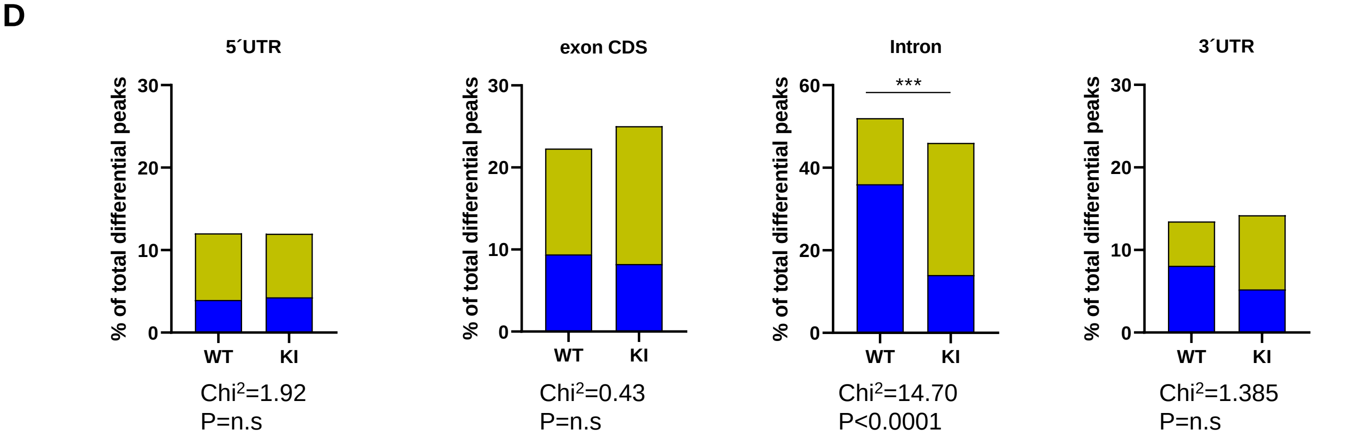

Supplementary Figure 8. Distribution analysis of m6A peaks and correlation analysis between mRNA expression levels and m6A peak enrichment in WT and Hdh$^{+/Q111}$ (KI) mice. (**A**) Table showing comparison of differential mRNA expression with differential m6A peaks in 8 months old WT and KI mice (trained vs naive). (**B,C**) Correlation between the FC of mRNA abundance in trained mice relative to naive mice and the log2 ratio of m6A enrichment (trained vs naive) in WT (**B**) and KI (**C**) mice. Yellow, blue and grey dots represent downregulated peaks in trained mice, upregulated peaks, and non-differential peaks, respectively. P values were calculated from a Pearson´s product-moment correlation. Correlation between upregulated mRNA expression and m6A peaks is significant in both genotypes. (**D**) Differential distribution analysis of the down- and up-regulated m6A peaks (trained vs naive) between 8 months old WT and KI mice in the four transcript segments: 5’UTR, CDS exon, 3’UTR, intron. Behavioral training induces a higher percentage of upregulated m6A peaks in introns of WT mice when compared to KI mice, which in turn have a higher percentage of downregulated m6A peaks in introns. Data are shown as percentage of total differential peaks. Yellow represents downregulated peaks and blue represents upregulated peaks. Statistical analysis was performed using Chi^2. ***P < 0.001 compared with WT.

Supplementary Figure 8

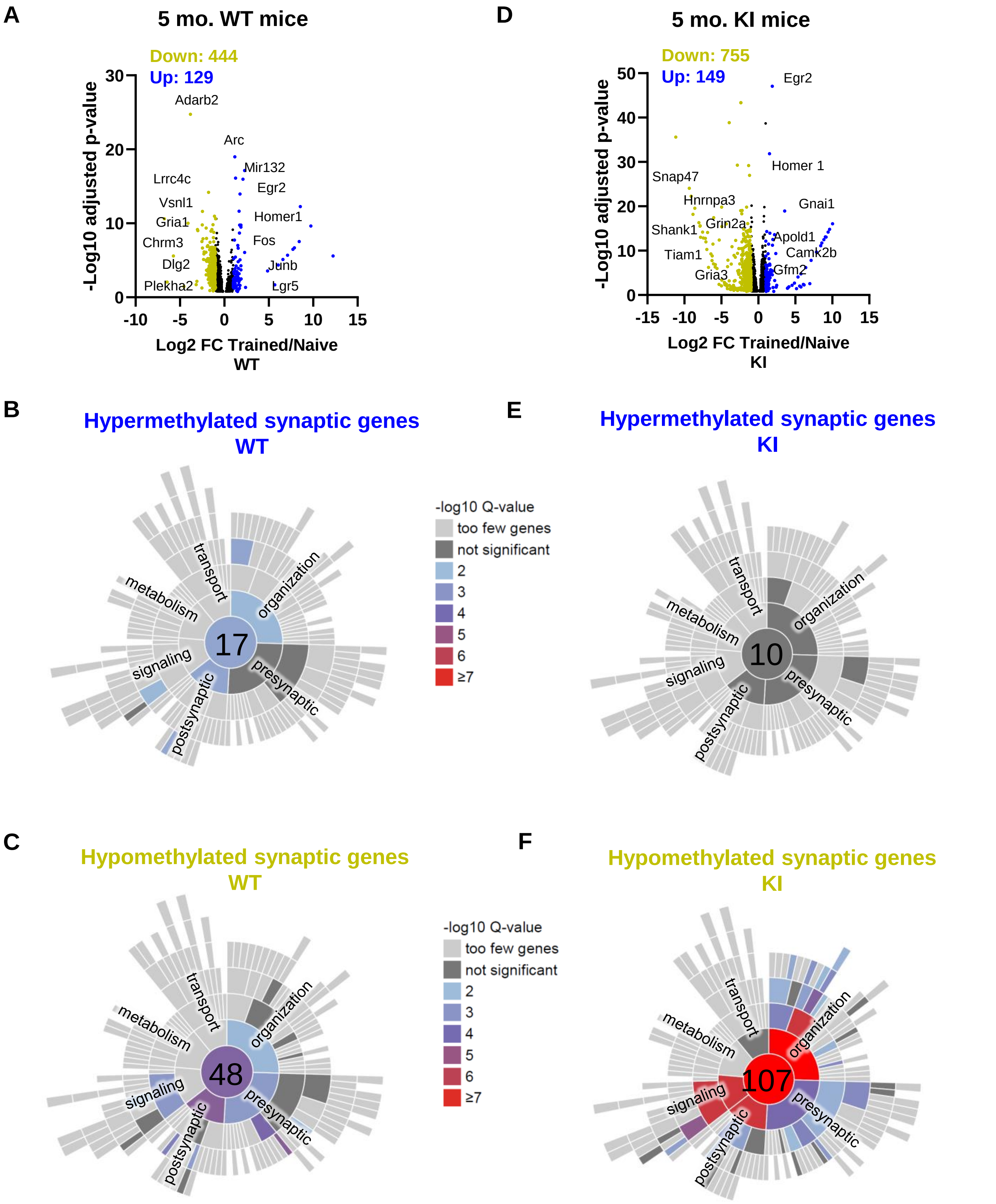

Supplementary Figure 9. Pre-symptomatic Hdh$^{+/Q111}$ (KI) mice show aberrant m6A demethylation of synaptic genes in response to behavioral training. (**A**) Volcano plot of the differentially methylated peaks between 5 months old naive and trained conditions in WT animals. Names of representative genes are indicated. (**B,C**) Sunburst plots for synaptic GO of differentially hypermethylated (**B**) and hypomethylated (**C**) genes in response to the OLT training task in WT mice. (**D**) Volcano plot of the differentially methylated peaks between 5 months old naive and trained conditions in KI animals. (**E,F**) Sunburst plots for synaptic GO of differentially hypermethylated (**E**) and hypomethylated (**F**) genes in response to the OLT training task in KI mice. In response to the behavioral training task, KI mice show increased number of hypomethylated genes enriched in synaptic terms. Numbers in the center indicate number of genes enriched in synaptic terms. GO analysis was performed with the SynGo knowledgebase. Screening threshold: adjusted p-value <0.05 and log2 fold change > 1 or < -1.

Supplementary Figure 9

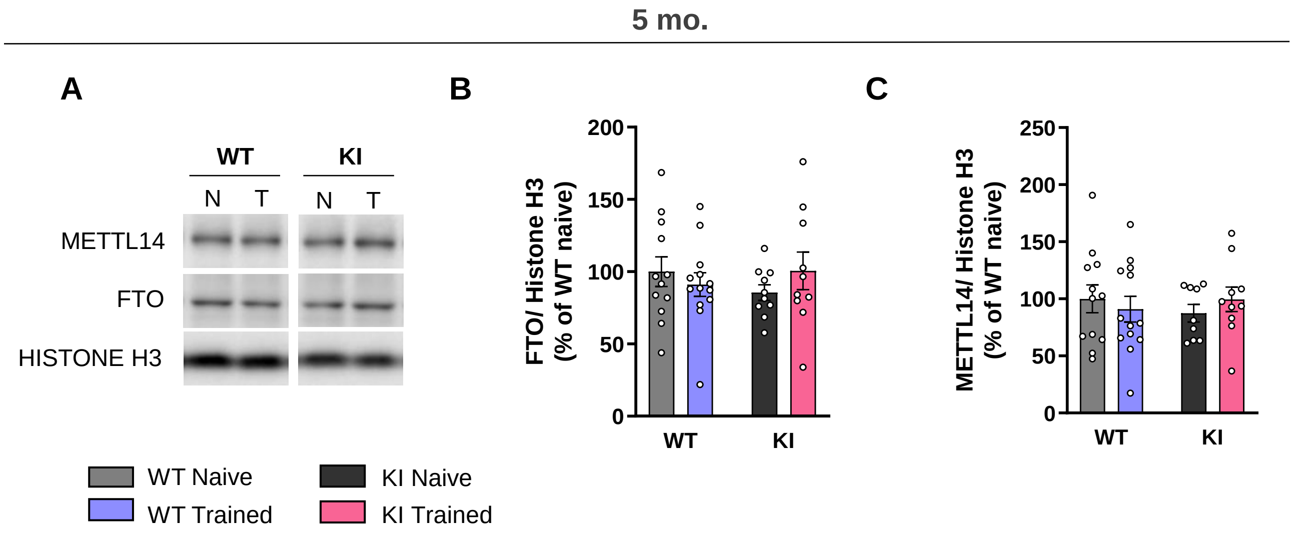

Supplementary Figure 10. Protein levels of the m6A-modifying machinery in the nucleus of 5 months old Hdh$^{+/Q111}$ (KI) mice. (**A**) Representative Western Blot for nuclear METTL14, FTO and Histone H3 in the hippocampus of 5 months old mice. (**B,C**) Histograms showing the relative nuclear protein levels of FTO (**B**) and METTL14 (**C**) in hippocampal extracts of 5 months old WT and KI mice subjected to the training in the OLT (n=9-13 animals/condition). Nuclear fraction was normalized to Histone H3. Two-way ANOVA with Tukey's multiple comparisons test. Data are presented as mean ± SEM. N, naive; T, trained.

Supplementary Figure 10

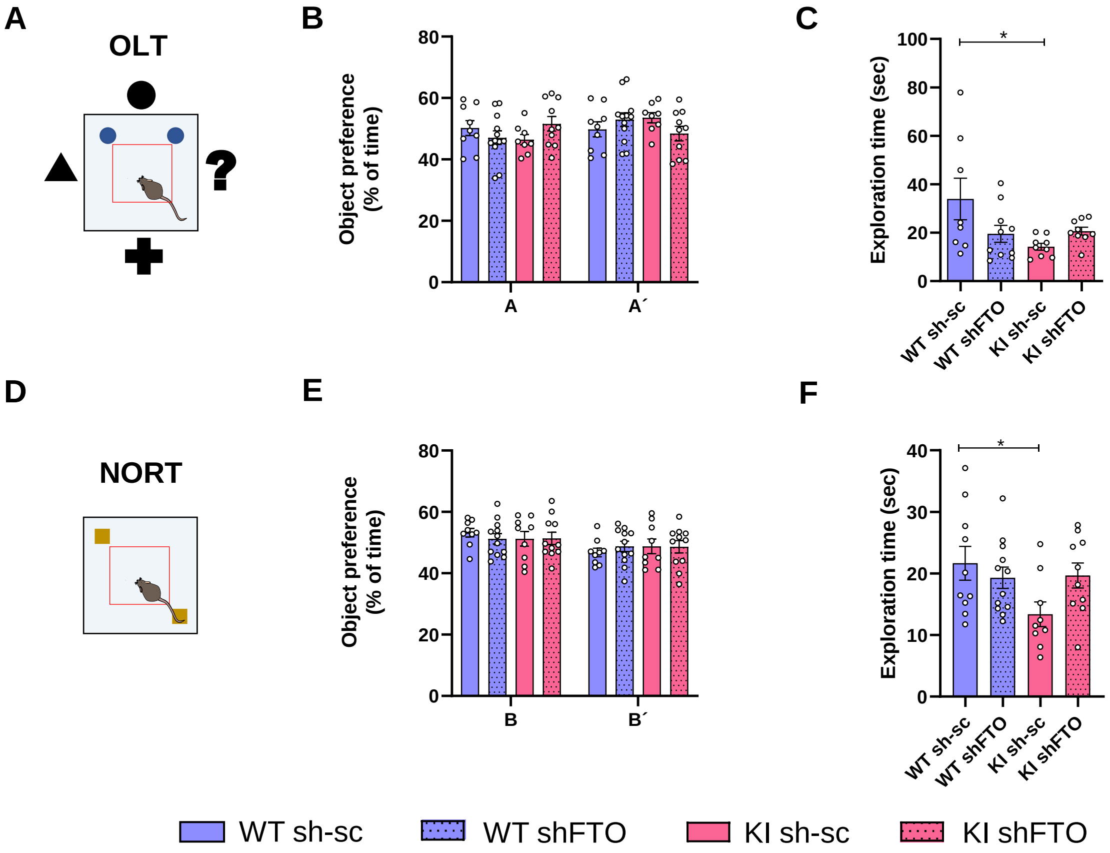

Supplementary Figure 11. Behavioral assessment of WT and Hdh$^{+/Q111}$ (KI) mice during the OLT and NORT training session. In the Novel object location task (**A**) and Novel object recognition task (**D**) all mice were first habituated to the open field arena and ambient conditions during two consecutive days and then subjected to a training session in the arena in the presence of two similar objects (A and A´ (OLT) and B and B´ (NORT)). (**B,E**) Percentage of time exploring objects for WT sh-sc, WT shFTO, KI sh-sc and KI shFTO at 8 months of age shows that all genotypes similarly explored both objects in the OLT (**B**) and NORT (**E**) training sessions, indicating no object or place preferences in the animals. (**C,F**) Exploration time (sec) during the training session of OLT (**C**) and NORT (**F**). Mutant KI sh-sc mice showed significantly less exploration time. Two-way ANOVA with Tukey's multiple comparisons test; Two-way ANOVA for the OLT training session reported a significant interaction effect ($F(1,32)=5.554$, $p=0.0247$) and a significant genotype effect ($F(1,32)=4.430$, $p=0.0433$). Two-way ANOVA for the NORT training session reported a significant interaction effect ($F(1, 36)=5.512$, $p=0.0245$). *$P < 0.05$ compared with WT sh-sc mice. Data are presented as mean ± SEM (n=9-12 per genotype). sh-sc, scramble shRNA.
